# Predicting the Outbreak Risks and Inflection Points of COVID‐19 Pandemic with Classic Ecological Theories

**DOI:** 10.1002/advs.202001530

**Published:** 2020-09-24

**Authors:** Zhanshan (Sam) Ma

**Affiliations:** ^1^ Computational Biology and Medical Ecology Lab State Key Laboratory of Genetic Resources and Evolution Kunming Institute of Zoology Chinese Academy of Sciences Kunming 650223 China; ^2^ Center for Excellence in Animal Evolution and Genetics Chinese Academy of Sciences Kunming 650223 China

**Keywords:** Allee effects, coronaviruses, infection aggregation critical threshold, outbreak inflection (turning or tipping) points, ratio of migrational infections to local contagions |COVID‐19 (coronavirus disease)

## Abstract

Predicting the outbreak risks and/or the inflection (turning or tipping) points of COVID‐19 can be rather challenging. Here, it is addressed by modeling and simulation approaches guided by classic ecological theories and by treating the COVID‐19 pandemic as a metapopulation dynamics problem. Three classic ecological theories are harnessed, including TPL (Taylor’s power‐law) and Ma’s population aggregation critical density (PACD) for spatiotemporal aggregation/stability scaling, approximating virus metapopulation dynamics with Hubbell’s neutral theory, and Ma’s diversity‐time relationship adapted for the infection−time relationship. Fisher‐Information for detecting critical transitions and tipping points are also attempted. It is discovered that: (i) TPL aggregation/stability scaling parameter (*b* > 2), being significantly higher than the *b*‐values of most macrobial and microbial species including SARS, may interpret the chaotic pandemic of COVID‐19. (ii) The infection aggregation critical threshold (*M*_0_) adapted from PACD varies with time (outbreak‐stage), space (region) and public‐health interventions. Exceeding *M*_0_, local contagions may become aggregated and connected regionally, leading to epidemic/pandemic. (iii) The ratio of fundamental dispersal to contagion numbers can gauge the relative importance between local contagions vs. regional migrations in spreading infections. (iv) The inflection (turning) points, pair of maximal infection number and corresponding time, are successfully predicted in more than 80% of Chinese provinces and 68 countries worldwide, with a precision >80% generally.

## Graphical abstract

Predicting the outbreak risks and/or the inflection (turning or tipping) points of newly emerging infectious diseases such as COVID‐19 can be rather challenging. Here, the challenge is addressed by modeling and simulation approaches guided by classic ecological theories and by treating the COVID‐19 pandemic as a spatiotemporal dynamics problem on the metapopulation scale.

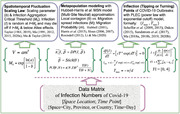

## 1 Introduction

**Figure 1:**
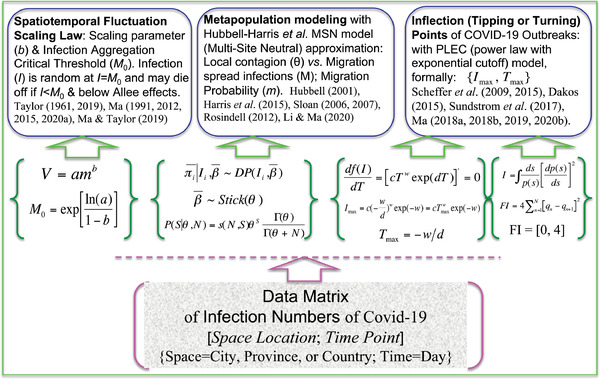
The three objectives and supporting approaches for this study.

The present study is aimed to address three important questions regarding the outbreaks of COVID‐19 (coronavirus‐infected pneumonia disease 2019) (https://www.who.int/emergencies/diseases/novel-coronavirus-2019)^[^ ^1^, ^2^ ^]^ by leveraging the insights from three classic ecological laws (models). Specifically, our objectives include (**Figure** **1**): i) investigating the outbreak risk of COVID‐19 by using Taylor's power law (TPL) and Ma's population aggregation critical density (PACD) for measuring the spatiotemporal aggregation scaling;^[^ ^3^, ^4^, ^5^, ^6^ ^]^ ii) evaluating the relative importance of local contagion versus regional migration in spreading the infections by approximating the metapopulation of COVID‐19 infections with Hubbell's unified neutral theory of biodiversity (UNTB) implemented with Harris et al. multisite neutral model (MSN);^[^ ^7^, ^8^ ^]^ iii) predicting the inflection (turning or tipping) points of outbreaks by establishing infection–time relationship (ITR) inspired by classic species–time relationship (STR) and Ma's diversity–time relationship (DTR) in biogeography.^[^ ^9^, ^10^ ^]^

First, the spatial and/or temporal distributions of many biological populations including microbes and humans follows TPL,^[^ ^3^, ^4^, ^11^, ^12^, ^13^ ^]^ and recent studies have also revealed its applicability at the community scale,^[^ ^4^, ^6^, ^14^, ^15^ ^]^ even beyond biology and ecology.^[^ ^16^, ^17^, ^18^, ^19^, ^20^ ^]^ Before this study, there have already been its applications to the analyses of spatial aggregation (variation) of human population,^[^ ^21^ ^]^ human mortality,^[^ ^22^ ^]^ and epidemiology.^[^ ^23^ ^]^ We therefore postulate that TPL should also be applicable to the spatiotemporal aggregation/fluctuation analyses of coronavirus infections such as the still ongoing COVID‐19 pandemic. In theory, there are three fundamental spatial distribution patterns for biological populations: aggregated, random, and uniform. In reality, the spatial distribution of population is usually a *continuum* consisting of the three patterns, depending on population abundance (density).^[^ ^5^, ^6^, ^17^ ^]^ The uniform and aggregated distributions are on both ends of the continuum, with random distribution being a transition point between the both. When population abundance (such as virus infections) crosses the so‐termed PACD, defined by Ma,^[^ ^5^, ^6^ ^]^ its spatial distribution may turn into aggregated or uniform depending on the sign of TPL scaling parameter, being random at the PACD.^[^ ^5^, ^6^ ^]^ The phenomenon is similar to percolation process that has been applied to describe the spread (expansion) of forest fire or outbreak of infectious diseases.^[^ ^20^, ^23^ ^]^ In forest fire, isolated local fire spots (patches) may suddenly become connected and then the whole regional forest may burn to ground. Similarly, in epidemiology, when local community contagions (which might be random or lightly aggregated) reach a critical threshold, the infections may become highly aggregated and connected regionally, ultimately leading to an epidemic or even pandemic globally.

Second, while TPL can be harnessed to analyze the spatiotemporal fluctuations of coronaviruses infections, we also aim to understand the spread of the virus infections from both local contagion (endemic) and regional (epidemic and pandemic) migration perspectives. Nevertheless, this can be rather challenging given the lack of controlled experimental data, which is ethically infeasible to collect obviously. In principle, all of the infections existing globally constitute a metapopulation of people infected by the coronavirus, but constructing standard epidemiological models^[^ ^24^, ^25^ ^]^ with existing data can be rather challenging. We realized that Hubbell's neutral theory of biodiversity,^[^ ^7^ ^]^ which is one of the four major metacommunity paradigms (the other three include species sorting, mass effect, and patch dynamics),^[^ ^26^, ^27^ ^]^ can be adapted to approximate the metapopulation dynamics. This approximation allows us to obtain, to the minimum, an educated guess for the parameters regarding the local contagion spreading versus regional (global) dispersal (migration) spreading of the infections.

Third, investigating the inflection (turning) points of infectious disease outbreaks falls under the domain of tipping points (TPs) and critical transitions.^[^ ^28^, ^29^, ^30^, ^31^ ^]^ Predicting the inflection points, also known as the tipping points or turning points, turned out to be notoriously difficult. In theory, it has been suggested that even without fully mechanistic understanding of the underlying critical transitions, it is still possible to infer generic features or early warning signals (EWS) of approaching a tipping point from time series or spatial pattern data. Theory proposes that at bifurcation points (i.e., tipping points), the stability of an equilibrium changes and the dominant real eigenvalue becomes zero. Consequently, the recovery rate from disturbance should go zero when approaching such bifurcation or the return time (measure of resilience) should be infinity.^[^ ^28^ ^]^ In practice, the recovery rate from disturbance may be an indicator of the distance to a tipping point, and its manifestation, the critical slow down, offers important EWS for approaching a tipping point. However, in a pre‐experiment analysis of the existing datasets of COVID‐19 infections, we failed to detect any EWS for tipping points, as demonstrated in Table S10 in the Supporting Information. We then also tried some simple epidemiological modeling approaches such as simple logistic model^[^ ^32^ ^]^ but still failed to achieve any satisfactory results.

Inspired by the exponential‐like growth of infections leading to peaks, which seems to be a hallmark of many infectious disease outbreaks including COVID‐19 and severe acute respiratory syndrome (SARS),^[^ ^1^, ^2^, ^25^, ^33^ ^]^ we try the PLEC (power law with exponential cutoff) model for detecting the inflection points in the time series data of COVID‐19. The PLEC model (function) starts with power function increase (near exponential growth usually) and it has an exponential decay term that acts as taper‐off parameter to eventually overwhelm the power law behavior at very large value of independent variable. It was previously used to describe the classic species‐area relationship (SAR) in biogeography^[^ ^34^, ^35^ ^]^ and later used for extending the classic SAR and STR to general diversity–area relationship (DAR) and DTR by Ma.^[^ ^9^, ^10^, ^36^ ^]^ Furthermore, Ma^[^ ^9^, ^10^, ^36^ ^]^ derived the formula for estimating the maximal accrual diversity in space (area), time, and/or spatiotemporal settings, which may also be used to estimate *potential* diversity (also known as “*dark*” diversity). The potential or dark diversity refers to the biodiversity that take into accounts the contribution of species that are absent locally but potentially present in regional or global species pool. This situation of potential species migration from regional (metacommunity) to local community is not unlike the COVID‐19 infections via dispersal (migration) such as travelers of virus‐infected individuals. We postulate that the PLEC model can be useful for modeling the ITR—the relationship between the cumulative number of infections and time points (e.g., day 1, 2, ……, *N*). If the PLEC model successfully fits to the ITR datasets, we further postulate that the maximal accrual diversity or potential diversity and corresponding time point may be translated into an approach for estimating the *maximal infection number* (*I*_max_) of an outbreak and the corresponding time point (when *I*_max_ is first passed or the *first passage time*) as estimate of inflection time point (*T*_max_). Together the pair of {*I*_max_, *T*_max_} is defined as the inflection (turning) point of a disease outbreak. Figure 1 is drawn to illustrate the three objectives of this study and the mathematical models harnessed to achieve the objectives as well as their theoretical and practical implications.

## 2 Results

### 2.1 Spatiotemporal Aggregation/Stability Scaling of Coronaviruses Infections—The Infection Critical Threshold and Insights for Outbreak Risks

**Figure 2:**
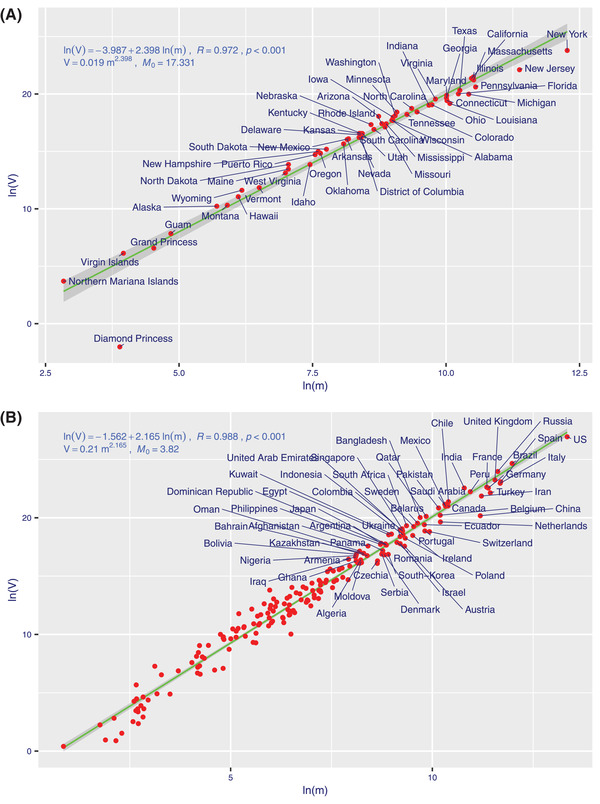
The TPL (Taylor's power law) model fitted to the cumulative infections of COVID‐19: A) in the USA and B) in the World.

From Tables S1–S5 in the Supporting Information, we obtain the following findings (also *see* **Figure** **2** and Figure S1, Supporting Information):

i) The spatiotemporal aggregation (stability) scaling of COVID‐19 infections followed TPL (Taylor's power law) at all scales (schemes) tested, including world‐wide, country‐wide, province (state, city)‐wide, cumulative, and daily incremental infections (as evidenced by the *p*‐value < 0.001 or *R* > 0.95 from the TPL fittings). We tested the combinations of primary versus alternative fitting schemes, fitted to cumulative versus daily incremental infections. While the fittings of TPLs with all four combinations passed statistical tests (all *p*‐values < 0.001, Tables S1 and S2, Supporting Information), we found that the TPL model based on primary fitting scheme with cumulative infections is the most appropriate for our objective. With the primary scheme, *V–m* (variance–mean) pairs, which are computed by averaging across *N*_Time_ time series points, are regressed over *N*_space_ spatial points (the alternative scheme used the opposite computation scheme as detailed in the Supporting Information).
ii) It was discovered that the infections of COVID‐19, like populations of other organisms, follow seeming universal power law. This implies that the infection of the novel coronavirus is highly aggregated (contagious) spatially and temporally its outbreak (spreading) is chaotically unstable in general, as indicated by the *b*‐values exceeding *2* (for cumulative infections) or exceeding *1* (for incremental infections) (Tables S1 and S2, Supporting Information). It was also found that COVID‐19 has a significantly larger *b*‐value than SARS (Table S5, Supporting Information) (worldwide *b*‐value = 2.165 vs 2.020) (*P*‐value = 0.049).
iii) The PACD or *M*_0_ (i.e., renamed as “*infection aggregation critical threshold*”) (Tables S1 and S2, Supporting Information) seems to depend on disease kinds (COVID‐19 or SARS), space (regions), and time (stage of disease outbreak), as indicated by the randomization test results (Tables S3–S5, Supporting Information). Given that *M*_0_ measures the infection threshold at which infections are random (or follow Poisson distribution), a lower *M*_0_ may indicate a lower infection “tolerance” level since crossing the level of *M*_0_ may signal the highly aggregated (unstable) infections. For example (*see* Table S1, Supporting Information), based on the data until June 12, *M*_0_ = 16.751 for COVID‐19 in China, *M*_0_ = 3.802 for COVID‐19 worldwide, *M*_0_ = 17.331 for COVID‐19 in the US may suggest that the infection tolerance level of COVID‐19 in China and US are actually higher than those in the rest of the world. Hubei province of China (the previous epicenter of the outbreak in China) has the highest *M*_0_ = 31.99, indicating the highest tolerance level for outbreak risk.

It should be noted that *M*_0_ is a parameter without *specific, explicit or fixed* occurrence‐time label. In other words, different regions (e.g., China and US) may have the same or similar *M*_0_ at different time points. In such cases, cautions must be taken when attempting to compare the values of *M*_0_. In the case of COVID‐19 pandemic, we can treat it as a threshold at which the infections are random, rather than *aggregated* or *uniform*. If proper measures are taken, the infections may be suppressed under *M*_0_ (uniform or constant) and may die off (ultimately). Otherwise, the infections may become *aggregated*, leading to an outbreak. Therefore, one may imagine that if the current infection level in the US *were* controlled to or under *M*_0_, it would have similar outbreak risks as in China (which is currently under *M*_0_). So this is an asynchronous or time‐shifted comparison, and in reality, both places are not comparable at this time. In addition, the notion of “the higher *M*_0_ is, the lower risk is” is actually the “the higher *M*_0_, the higher tolerance level to infections is” in terms of the *threshold* crossing to aggregated infections that preceded outbreaks or pandemic.

Table S1 in the Supporting Information also listed the TPL parameters fitted to the infection data of Hubei province of China, and China nationally until April 10. Compared with their counterparts until June 12, the fluctuation scaling parameter *b* was nearly invariant (2.254 vs 2.259 for Hubei province and 2.192 vs 2.153 for China nationally). However, the infection aggregation critical threshold (*M*_0_) nearly doubled for both Hubei and China during the period. Given that the new infections in China during the period of April 10 to June 12 are only 1.6% (or 1349), the comparisons of the TPL parameters (*b* and *M*_0_) suggested two interesting points. First, the spatiotemporal scaling parameter (*b*) is invariant with time, even when the epidemic dies off. Second, it might be the case that population (herd) immunity (in terms of the tolerance level for infection outbreak) is up after experiencing an epidemic and it is emphasized that this notion of raised immunity level is largely a speculation without additional supporting evidence.

iv) Note that *M*_0_ is the *mean* infection level (aggregation critical threshold); therefore, it can be more meaningful to convert it into absolute value from a practical perspective. For example, according to the data computed until April 10, *M*_0_ = 9.525 for COVID‐19 in China, when converted to total national infection level, would be 9.525 × 34 (provinces) = 323 nationally. This threshold number may suggest that when the number of total daily infections nationally is at this level, the infections are random, rather than aggregated. When infections exceed this threshold level, the infections should be aggregated spatially and highly unstable temporally. When infections are below this threshold level, the infection should be uniform spatially and rather stable (following a uniform distribution statistically) or may even die off if the threshold level coincides with the level of Allee effects. Since the level of Allee effects is still unknown, whether or not the infections below *M*_0_ will die off is still an open question. From April 10 to June 12, the daily incremental infections numbers in China have been under 100 << 323 (computed previously based on *M*_0_), the fact that no new outbreak occurred in China during the period casts a piece of supporting evidence on our previous interpretations on *M*_0_. In fact, the *M*_0_ rose to 16.751 in June 12, which suggested that the tolerance level for outbreak in China was even higher than in April (16.751 × 34 provinces = 570 nationally).

### 2.2 Approximating Metapopulation Dynamics of Coronavirus Infections with Metacommunity Model—Relative Importance of Regional Migrations versus Local Contagions in Spreading Infections

**Figure 3:**
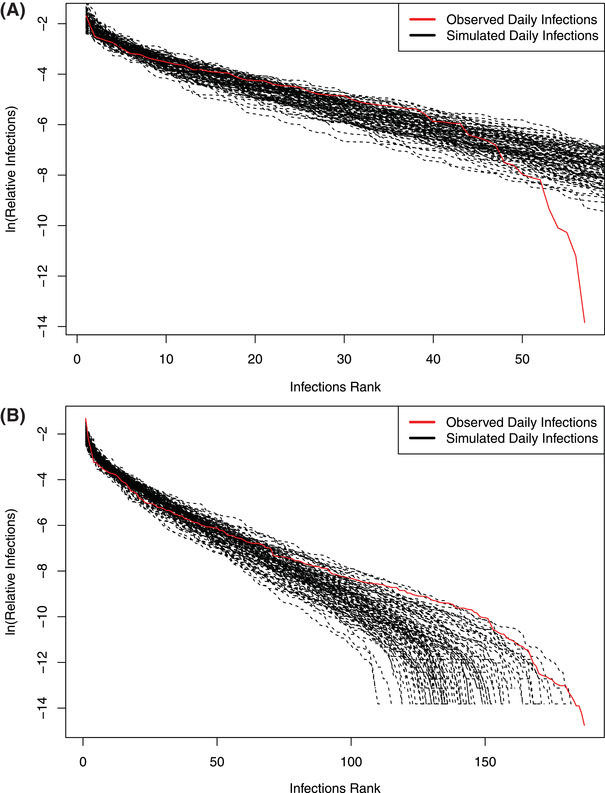
Approximating the metapopulation of daily incremental COVID‐19 infections with Hubbell's UNTB for metacommunity, implemented with Harris et al. HDP‐MSN model:^[^ ^7^, ^8^ ^]^ A) with datasets of the USA and B) with datasets of the world.

**Figure 4:**
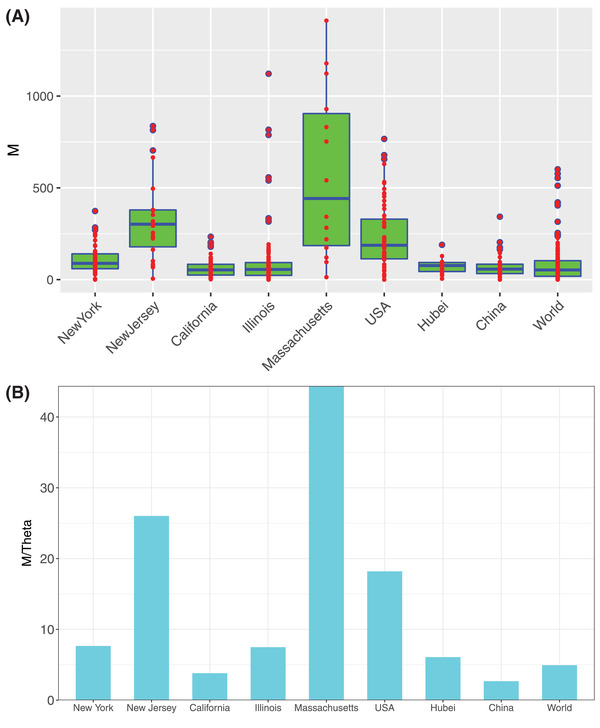
Parameters from approximating metapopulation dynamics with metacommunity neutral modeling: A) The box plots showing the fundamental dispersal number (*M*) computed from fitting the MSN model to the COVID‐19 infections. Three standard summary numbers (statistics) of the parameters (*M*), including the first quartile (lower edge of the rectangle), median (the inside segment), and third quartile (upper edge of the rectangle) were displayed, respectively. The interquartile range (IQR) (showing the range of variation) is displayed by the height of the box and the median shows the typical value. Outliers (<3 × IQR or >3 × IQR) are displayed outside the box. B) The *M*/*θ* (ratio of *fundamental dispersal number* to *fundamental contagion number*) as an indicator for gauging the relative importance of regional migration versus local contagion in spreading COVID‐19 infections

As expected, all datasets passed the neutrality tests of the hierarchical Dirichlet process (HDP)‐MSN model as indicated by the *p*‐value > 0.05 (Table S6, Supporting Information). From **Figures** **3** and **4** and Table S6 and Figure S2 in the Supporting Information, we obtain the following findings:

The fundamental biodiversity number (*θ*) measures the “speciation rate” (in metacommunity theory), which indicates the infection level from local contagion, and we rename it *fundamental contagion number* in this study. The fundamental dispersal (migration) number (*M*) measures the “migration rate” (in metacommunity theory), which indicates the infection spreading from regional migration. We use the ratio of *Q *= *M*/*θ* as a measure of the relative importance of “infection spreading via regional migration” versus “infection spreading via local contagion,” with larger *Q* indicating higher migration role and smaller *Q* indicating higher local contagion. The *Q*‐value in the New Jersey (26.022), New York (7.659), Massachusetts (44.364), and USA (18.192) seem significantly higher than those in the world (4.936) including Hubei of China (2.677) and China nationally (6.079).

Compared with the early results of China until April 10, the level of *Q* for Hubei province of China slightly declined (6.84 vs 6.079), but the national level of *Q* in China declined significantly from April 10 to June 12 (6.279 vs 2.677). Given that Hubei was the previous epicenter of the outbreak in China, these results should be expected, i.e.*, Q* (the role of migrational spreading) with little change in the epicenter but significant decline outside the epicenter after experiencing an outbreak.

We argue that the ratio *Q* depends on time (epidemic stages), space (regions), and disease‐kinds (COVID‐19 or SARS), and perhaps most importantly, public‐health interventions such as quarantines, social distancing, and/or mobility control (Figure 4B). The US states of Massachusetts and New Jersey exhibited the highest *Q*‐value (*Q = *44.365 and 26.022, respectively) and seemed to be mostly influenced by outside inputs from surrounding regions. The fundamental dispersal number (*M*) measures the average migration rate between regional (local) communities. Therefore, the differences in *M* among different regions (countries) may reflect the effects in mobility control (Figure 4A).

Compared with COVID‐19, SARS appeared to exhibit a different pattern of the relative importance of migration versus local contagion in spreading the infections, i.e., *Q* < 1 for SARS. We postulate that this difference should, at least partially, explain the extremely catastrophic nature of COVID‐19 pandemics compared with SARS.

### 2.3 PLEC‐ITR Model for Predicting the Inflection Points of COVID‐19 Outbreaks

#### 2.3.1 Feasibility Demonstration

**Figure 5:**
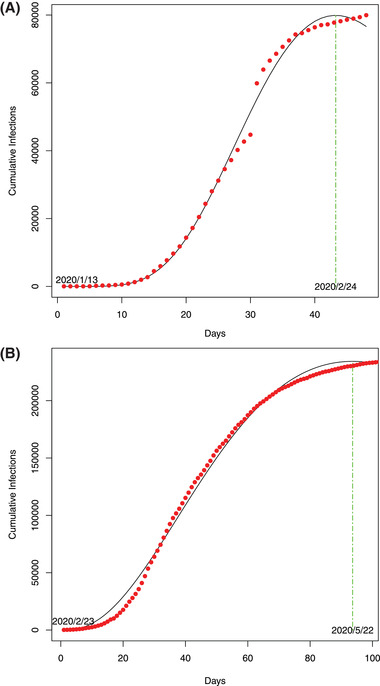
A) The ITR‐PLEC model fitted to the COVID‐19 infections: A) in China (*X*‐axis: Day 1 = January 13, 2020): the dots represent for observed infection numbers and the curve for the ITR‐PLEC model and B) in Italy (*X*‐axis: Day 1 = February 23, when the initial cumulative infection number >100): the dots represent for the observed infection numbers and the curve for the ITR‐PLEC model.

Our attempts to predict the inflection (turning) points by modeling the ITR with PLEC model are divided into two parts. First, we used the COVID‐19 infection datasets of China to demonstrate the feasibility of the approach given that the outbreak in China has apparently passed the inflection points in the early March (**Figure** **5**A). This part has been reported in a preprint of this paper,^[^ ^33^ ^]^ from which we summarize the results and verification methods in Tables S7 and S8 in the Supporting Information, and primary findings as follows:

i) Regarding the estimation of the *inflection time point* (*T*_max_), the PLEC‐ITR model correctly estimated the occurrences of *T*_max_ in 88% (15 out of 17 cities) cities of Hubei province of China and 85% (29 out of 34 provinces) at the provincial level in China. That is, on the predicted date of *T*_max_, the observed infection number is within the 95% interval of *I*_max_ (*maximal infection number*).
ii) Regarding the *maximal infection number* (*I*_max_), the success rates for the 17 cities in Hubei provinces were 88%, 100%, and 100% respectively, in terms of the observed infection numbers at the dates of *T*_max_, March 6 and March 12, respectively. Regarding *I*_max_ for the 34 provinces in China, the *success rates* were 85%, 85%, and 82% respectively, in terms of the observed infection numbers at the dates of *T*_max_, March 6 and March 12, respectively. The “success” means that the error rate is under 5% or the *Precision rate* (=1 − *Error rate*) is above 95%.
iii) The above two categories of results showed the *success rates* in predicting the inflection points {*T*_max,_ *I*_max_}, a third metric to show the feasibility of the approach is the average error rate (*Error*%) or precision rate (*Precision*% = 1 − *Error*%). As shown in Table S8 in the Supporting Information, the average *error level* for the 17 cities in the Hubei province of China was 1.9% (averaged from 17 cities over three verification dates) or 98.1% in terms of the *precision* level. The average error level for the 32 provinces (excluding two failed models) over three verification dates in China was 3.2% or 96.8% in terms of the *precision* level.

#### 2.3.2 Application to Worldwide Infection Data

We fitted the PLEC‐ITR model to the worldwide infection datasets (until June 12) for each country (recognized by world health organization, WHO). To increase the chance of successful fitting, rather than using the very initial date of recording an infection case, we left truncated the dates with accumulated (initial) infection numbers less than 100. Obviously, the truncation has no effect on the validity of predictions, since the truncation operation only shifts the PLEC‐ITR curve along the *X*‐axis, not the *Y*‐axis that determines the maximal infection number (*I*_max_). As to the influence of the truncation on *T*_max_, it can be easily dealt with by simple manual adjustment of dates (Figure 5B).

Table S9 in the Supporting Information showed the “successful” fitting results of PLEC model to the infection datasets of 33 US States and 78 countries. Here with “successful” fitting, we mean that the inflection points (*I*_max_, *T*_max_) were successfully estimated. However, there were two US States (California and Nevada) and ten countries (such as Iran, Japan, and Singapore), the *P*‐values for some of their PLEC model parameter estimations exceeded >0.05 slightly. We decided to exclude them from successful modeling results for the sake of reliability in predicting the inflection points. That brings down the number of successful predictions of countries to 68. Among the 68 countries successfully modeled, the top three countries in terms of the current infection numbers (i.e., US, Brazil, and Russian) were still missing. This demonstrated the challenging of predictions, but it may simply indicate that the outbreaks in those countries are still from over. In fact, from our modeling predictions, it appears that there were only four US states and 38 countries, of which the inflection time points (*T*_max_) have actually passed until this writing.

From Table S9 in the Supporting Information, we summarize the following findings (also see **Figures** **7** and **8**). Note that all of the prediction evaluations were compared against the infection number on June 17^th^ (the day we stopped the analysis for finalizing the paper) since for most models the inflection points have not occurred yet until this writing. Therefore, we caution that the predictions could just be educated guesses in the current stage.

For the infection data of the US states (Figure 7A,B):

i) We were able to fit the PLEC model successfully to 31 US States, and the average *precision* level was 82.9% or 17.1% in terms of the *error* level.
ii) The average predicted *I*_max_ across 31 US states is 43 993. The average predicted *T*_max_ across 31 US states is 163 d, while the starting dates for building the PLEC model ranged from March 7 to March 30. Adding the *T*_max_ to the date range may indicate how far each US State is from the actual inflection time point.
iii) We define the *duration* (*D*_o_) of outbreak as the time period from the recording of the first case to the *first passage time* of *I*_max_ (i.e., the outbreak peak). If left truncation was used to build the PLEC model, then the truncated time period should be added to *T*_max_ to compute corresponding *D*_o_ for each US state, i.e., *D*_o_ =* T*_max_ + “Initial cutoff period with infections <100.”

Similarly, for the worldwide infections (Figure 8A,B):

iv) We were able to fit the PLEC model successfully to 68 countries, and the average *precision* level was 91.4%, or 8.6% in terms of the *error* level.
v) The average predicted *I*_max_ across 68 countries was 25 992. The average predicted *T*_max_ across the 68 countries was 95 d, while the starting dates for building the PLEC model ranged from January 18 to May 25 for the 68 countries. Note that the averages were computed based on the infection data of those 68 countries with successful predictions only.

The reality that we could only successfully build predictive PLEC‐ITR models in less than 1/2 attempted cases (31 out of 58 US States as well as 68 out of 187 countries) suggests that for many regions, the COVID‐19 outbreaks may still be in their early stages. One additional caution should be taken is that the previously discussed “averages” (such as the average *I*_max_ of 68 countries) are not very meaningful since the distributions of these parameters (*I*_max_, *T*_max_) are not Gaussian distribution, rather they satisfy the highly skewed power‐law statistical distribution. We tested the goodness‐of‐fitting of both Gaussian and power law distribution and found that both (*I*_max_, *T*_max_) did follow the power‐law distribution (*P*‐value = 0.688 and 0.999, respectively). The power law distribution has the so‐termed “no‐average” property, which states that the arithmetic average of the data satisfying the power law distribution is a rather poor indicator for the population (group) level.

## 3 Conclusions and Discussion

### 3.1 Spatiotemporal Fluctuation Scaling and Metapopulation Modeling

This sub‐section summarizes the conclusions regarding the first two objectives and discusses their implications. First, the coronavirus infections, including both COVID‐19 and SARS, appear to possess characteristic *aggregation* (*stability*) parameter (*b*) values, exceeding 2 with COVID‐19 possessing a significantly larger value. As suggested by the literature of TPL,^[^ ^4^ ^]^ when the TPL parameter (*b*) exceeds *2*, the metapopulation dynamics can be rather chaotic, indicating potentially chaotic behavior of coronavirus outbreaks. The TPL‐*b* values for both COVID‐19 and SARS are at the high end of the *b*‐value range in population ecology, compared with the *b*‐values of most microbial and/or macrobial species in existing literature, which are usually *b* < 2 (e.g., ref. ^[^ ^4^ ^]^). Furthermore, COVID‐19 with significantly higher *b*‐value than SARS (2.165 vs 2.020 worldwide) may explain the much more catastrophic behavior of COVID‐19 pandemic.

While TPL‐*b* appears rather stable or even invariant with time and/or space,^[^ ^37^ ^]^ the *infection aggregation critical threshold* (*M*_0_), which is the level at which infections are random (*V *= *m* or Poisson distribution), below which infections should be stabilized, and above which infections are highly aggregated and irregular, can depend on disease kinds (COVID‐19 or SARS), time (disease or outbreak stages), and space (regions). We postulate that *M*_0_ should also be influenced by public‐health interventions such as social distancing, quarantines, and travel restrictions. Therefore, *M*_0_ can be an important epidemiological parameter for evaluating the characteristics or states of disease outbreaks. A higher *M*_0_ for a region may signal higher *tolerance level* (or lower *outbreak risk*), but we caution that the terminology such as “tolerance” and “risk” is still ecological, rather than biomedical terms. Furthermore, the term “lower outbreak risk” may not have an explicit time label associated with it. This is because whether infections may die off or persist (even if the infection level is below *M*_0_) depends on an implicit assumption—whether or not *M*_0_ coincides or below the level of *Allee effect*.

Second, all datasets we studied readily passed the neutrality test with Harris et al. HDP‐MSN model and indicated that the approximation of the metapopulation with metacommunity in the case of coronavirus infections is feasible.^[^ ^8^ ^]^ Two parameters from the HDP‐MSN, i.e., fundamental contagion number (*θ*) and fundamental dispersal number (*M*), respectively measure the average infections from *local contagion* and average infections from “dispersal” (migration) from regional or global populations. In other words, *θ* measures the infection spreading level due to local contagion and *M* measures spreading level due to external migration. The ratio of *Q *= *M*/*θ* may be used as a measure of the relative importance of “infection spreading via migration” versus “infection spreading via local contagion” in spreading the infections, with larger *Q* indicating higher migration role and smaller *Q* indicating higher role of local contagion.

Third, both the TPL scaling law and metapopulation modeling may complement each other. We postulate that the three parameters: *M*_0_ (*infection aggregation critical threshold*), *M* (*fundamental dispersal number*), and *θ* (*fundamental contagion number*) may be used to evaluate the effectiveness of public‐health intervention measures. For example, an objective function in the form of (*M*_0_ < *αM* + *βθ*) may be used to evaluate various measures in achieving the objective—control the infections below *M*_0_. That is, by adopting public health interventions in the form of *α* (for manipulating the effects on *M* or infection‐spreading via dispersal) and/or *β* (for manipulating the effects on *θ* or infection‐spreading via local contagion), it is hoped that the infection level can be controlled below the *infection aggregation critical threshold* (*M*_0_). Then, to the minimum, the infections should be stabilized or become random, if not go extinct.

### 3.2 Prediction of Inflection (Turning) Points with PLEC‐ITR Model

The proposed PLEC‐ITR model successfully estimated the inflection points {*T*_max_ and *I*_max_} in more than 80% Chinese cities and provinces, and the error rates ranged between 0% and 18% or 82% and 100% in terms of the *precision* level. When the model was fitted to other countries, we succeeded in the fittings for 31 US states and 68 countries. It was also shown that once a model was successfully built, its prediction precision (>80% in general) is similar to what was demonstrated with Chinese datasets. This shows great promise of using PLEC‐ITR model as a general approach for predicting the inflections points, despite its apparent limitations that we summarize below:

First, we realized that there should be sufficiently long duration of data collections to build a successful PLEC‐ITR model. How long is sufficient? Based on our trial‐and‐error experiments, it appears that what matters most is the time between *T*_max_ and the ending date of data collection. In other words, it is the “forward” distance between the latest observation date and the emerging inflection (turning) point (date) that matters most. The positive side is that once the model building is successful, the model can be useful in general, given that the precision level can usually reach 80% or even near 100%. Therefore, a practical strategy can be to just try model building on daily basis until a success is achieved. *Practically*, even if one knows that tomorrow or even today is the inflection time point, the prediction is still valuable in our opinion, although such a prediction may not be considered as of much value in other kinds of predictions in other fields.

Another fix we adopted was to artificially left truncate the time series data of initial infections. For example, in the present study, we artificially removed the initial time points with cumulative infection number <100. This practice, as explained previously, does not influence the validity of the approach, although it significantly raises the success of model building. This phenomenon confirmed our previous observation: what matters most is the distance between emerging *T*_max_ and the latest date of recording the infection numbers. Overly long duration of initial points with negligible infection numbers may actually hurt the chance of successful model building.

**Figure 6:**
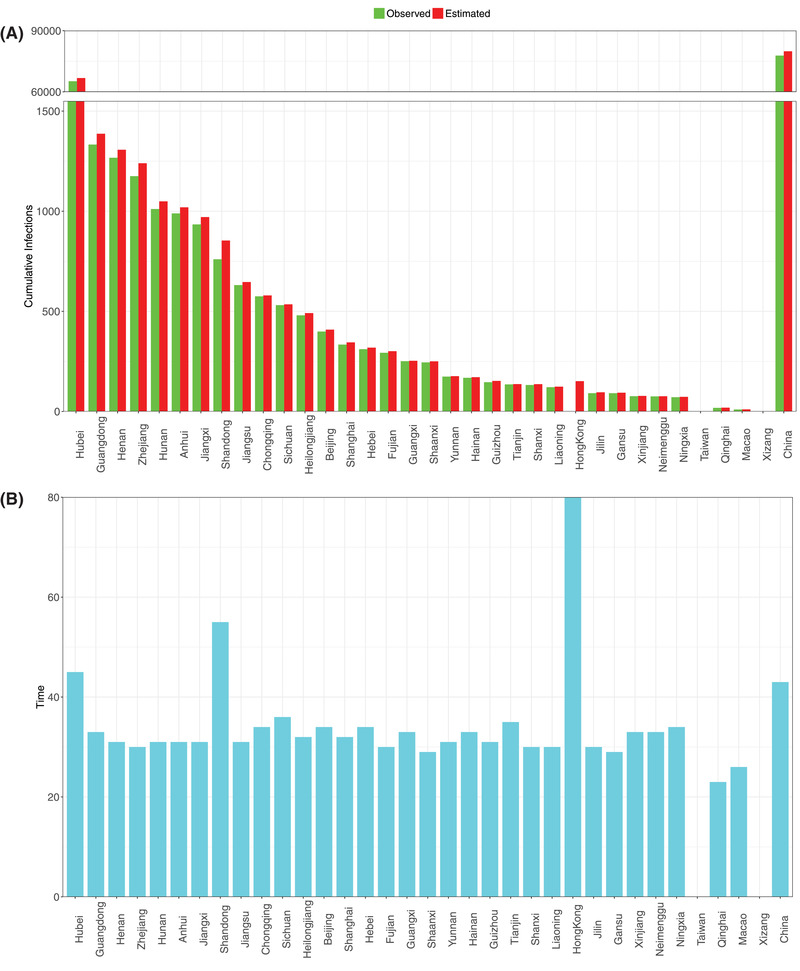
A) Comparisons of the observed and predicted maximal infection number (*I*_max_) for the 34 provinces of China; the rightmost bar represents for the maximal infection number (*I*_max_) of China nationally. B) The estimated inflection time point (*T*_max_) of COVID‐19 infections for 34 provinces of China; the rightmost bar represents for the inflection time point (*T*_max_) of China nationally: *Y*‐axis = Days, the starting date was January 11, 2020 (see Tables S7 and S8, Supporting Information, for the details).

**Figure 7:**
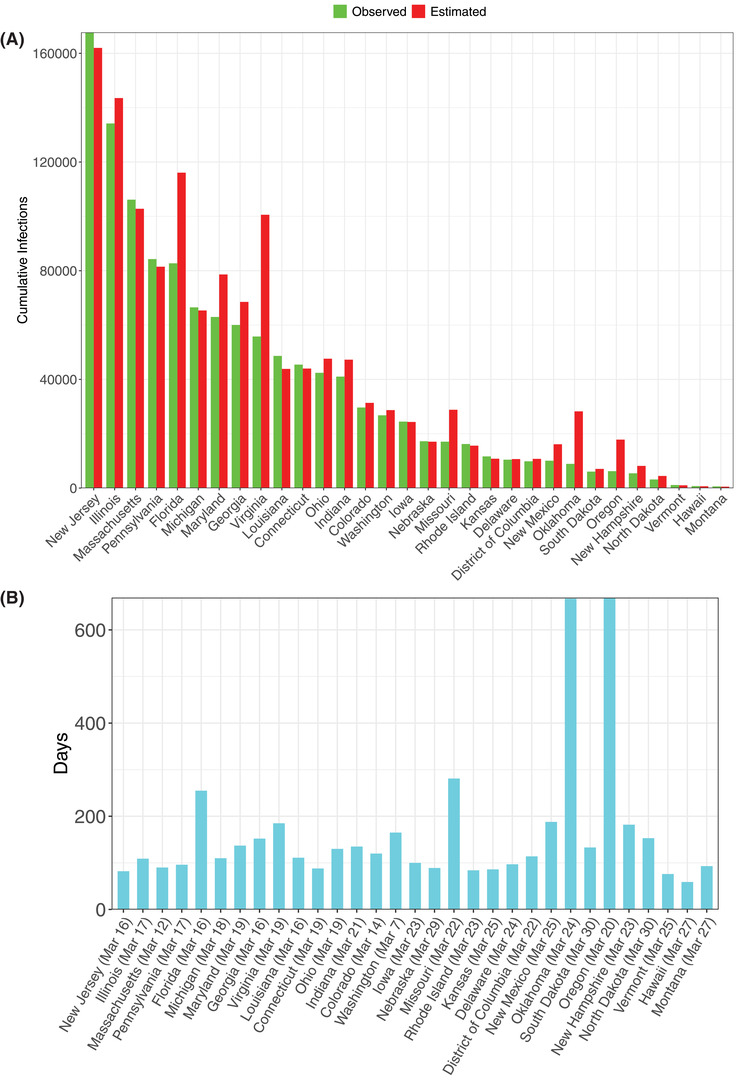
A) Comparisons of the observed and predicted maximal infection number (*I*max) of COVID‐19 infections for the 31 US States, for which the ITR‐PLEC models were fitted successfully. B) The estimated inflection time point (*T*max) of COVID‐19 infections for the 31 US States, for which the ITR‐PLEC models were fitted successfully (see Table S9, Supporting Information, for the details).

Second, it appears that small scale (city, province) modeling with PLEC‐ITR is more likely to succeed, as demonstrated in all city‐level successes in the Hubei Province (epicenter) of China (Figure S3, Supporting Information), followed by the provincial results in China (**Figure** **6**A,B), and the state‐level success in the USA (Figure 7A,B). These results showed limitations of the PLEC‐ITR model; nevertheless, the results from Fisher information (FI) technique for predicting tipping points may reveal the complexity of the problem, also further highlight the value of our PLEC‐ITR model. To save page space, the detection of tipping points for COVID‐19 outbreaks with Fisher information is presented in Table S10 in the Supporting Information.

**Figure 8:**
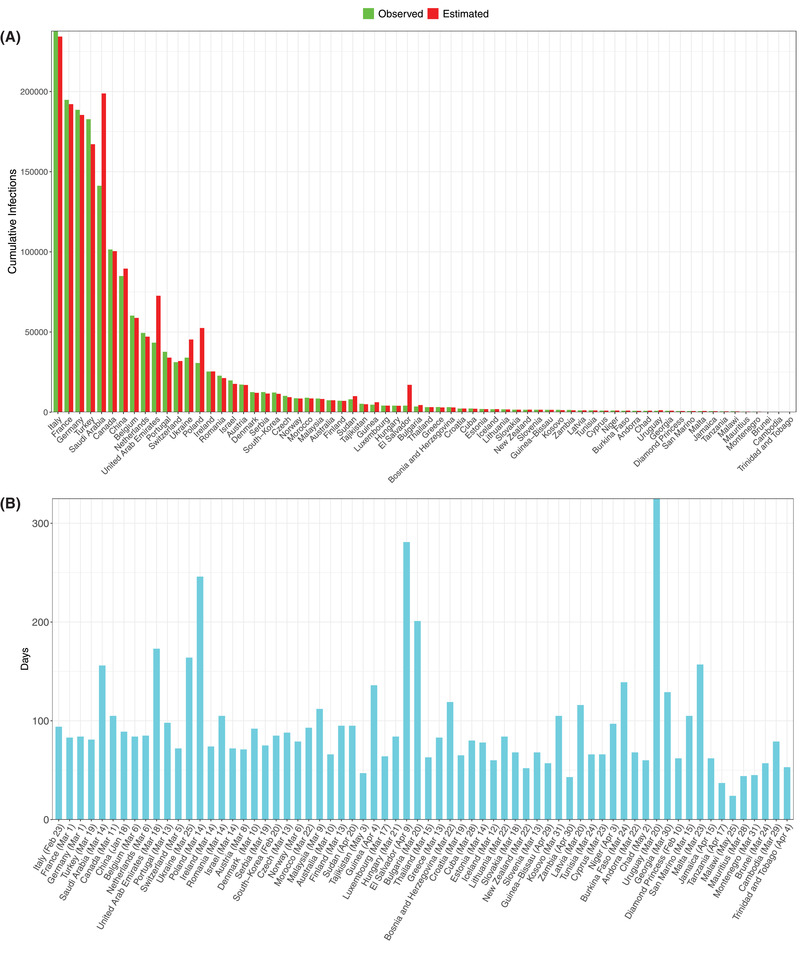
A) Comparisons of the observed and predicted maximal infection number (*I*_max_) of COVID‐19 infections for the 68 countries, for which the ITR‐PLEC models were fitted successfully. B) The estimated inflection time point (*T*_max_) of COVID‐19 infections for the 68 countries, for which the ITR‐PLEC models were fitted successfully (*X*‐axis showing the left truncated starting date; *Y*‐axis showing the days. Note that each country may have different staring dates due to the left truncation of days with initial cumulative infection number <100).

Third, our model is only applicable to unimodal (single peak) outbreak. Nevertheless, the PLEC modeling process may be repeated for the 2nd or a later round of outbreak. Finally, we would like to mention one potentially very important point regarding the prediction of inflection or tipping points, but is beyond the scope of this study, i.e., the critical influences of public‐health intervention/prevention measures on the occurrences of inflection (tipping) points. Such a study may only be feasible after the COVID‐19 pandemic is over worldwide and we hope the day will come sooner.

## 4 Experimental Section

### Datasets of COVID‐19 and SARS Infections

The worldwide, daily incremental, and cumulative infections of 2019‐novel coronavirus (COVID‐19) and SARS, respectively, were collected. For the datasets collected in China or US, the unit of data collections was set to Chinese provinces or US states, respectively. In addition, for the COVID‐19 infections, the datasets of 17 cities of Hubei province of China and 58 counties (or cities) of New York State were also collected, given that Hubei and New York were the apparent outbreak epicenters in China and US, respectively. For other countries or regions, the unit of data collections was set to country or region recognized by the WHO. The date range for collecting the SARS data was between March 17 and August 7, 2003 (136 d) and that for COVID‐19 was between January 11 and June 12, 2020 for the TPL analysis and metapopulation modeling. However, for inflection point predictions, an additional consideration has to evaluate the feasibility of the predictive models. Given that the COVID‐19 outbreak in China was largely over in early March, the infection datasets were used until February 29 in China for the purpose of testing the feasibility of predicting the inflection points. After verifying the feasibility of inflection prediction with Chinese data, for the rest of the world, the infection data from January 22 to June 12 were used for inflection point predictions. As a side note, for TPL and metapopulation modeling, there were two sets of results, corresponding to two different ending dates for data collections, i.e., April 10 and June 12. The earlier results were reported in the initially submitted version of this paper. Actually, the earliest results back to February were exhibited in the two preprints of this paper,^[^ ^33^, ^37^ ^]^ where the basic approaches were developed.

### TPL Spatiotemporal Aggregation Scaling Law for the Infections of Coronaviruses

Taylor discovered that the relationship between mean abundance (*m*) and corresponding variance (*V*) of biological populations follows the following power function^[^ ^3^ ^]^

(1) $$\begin{array}{c} V=am^{b} \end{array}$$

where *b* is termed *population aggregation* parameter and is thought to be species‐specific, and *a* is thought to be related to sampling schemes used to obtain the data. The relationship is known as TPL in literature.^[^ ^4^ ^]^ TPL can be converted into the following log‐linear model

(2) $$\begin{array}{c} \ln(V)=\ln(a)+b\ln(m) \end{array}$$

Note that the term *aggregation* at the population level can be considered as the counterpart of *heterogeneity* at the community level or as the counterpart of *stability* in temporal fluctuation (dynamics) modeling.^[^ ^6^, ^14^, ^15^, ^17^ ^]^ In the present study, the TPL analysis actually addresses the spatiotemporal aggregation (fluctuation or stability) scaling given that both temporal and spatial data were used.

To further harness the TPL parameters, Ma derived a third parameter (*M*_0_) for TPL or its extensions at the community scale,^[^ ^5^, ^6^, ^17^ ^]^ *population aggregation critical density* at the population scale or *community critical heterogeneity* at the community scale, which is in the form of

(3) $$\begin{array}{c} M_{0}=\exp\left[\ln\left(a\right)/\left(1-b\right)\right]\;\left(a>0,b\neq 1\right) \end{array}$$

where *a* and *b* are TPL parameter. *M*_0_ is the level of *mean* population abundance, i.e., the *mean* infection number of COVID‐19 or SARS in the case of this study, at which the spatial aggregation (or temporal fluctuation) of virus infections is random (following Poisson statistical distribution). When *m* > *M*_0_, the virus population (infection) distribution is highly aggregated on spatial scale; or in terms of time scale, the virus population (infection) fluctuation is highly irregular (or even chaotic). Translated into the terms of epidemiology, on spatial scale, the highly aggregated distribution of infections is an essential characteristic of infectious diseases in regional epidemics or in global pandemic. Similarly on time scale, highly irregular fluctuation of infections is another essential characteristic of infectious diseases in epidemic/pandemic states.

When *m* < *M*_0_, the infection distribution (fluctuation) is uniform (regular) and may follow the *uniform* statistical distribution. In this case, the infection fluctuation should be stabilized or might even die off. When *m *= *M*_0_, the infection is random and should follow Poisson distribution statistically, as introduced previously. In the context of this study, PACD or *M*_0_ is termed as *mean infection aggregation critical threshold* or simply *infection critical threshold*, which is similar to the classic Allee effects.^[^ ^38^ ^]^ Although the both are similar conceptually, it should be emphasized that they have different ecological interpretations. In particular, when *m *= *M*_0_, it means that the population spatial distribution or temporal fluctuation (stability) is random; when *m* < *M*_0_, the distribution or stability is regular or uniform. Whether or not the population may die off is uncertain. If *m* happens to coincide with the threshold of Allee effects, population (infections) may indeed die off.

### Approximating Metapopulation of Coronavirus Infections with Metacommunity Model: Hubbell's UNTB Implemented in Harris et al. HDP‐MSN Model^[^ ^7^, ^8^ ^]^

Hubbell's UNTB conceptually distinguishes between local community dynamics and metacommunity dynamics,^[^ ^7^ ^]^ both of which are assumed to be driven by similar neutral processes—stochastic drifts in species demography, local speciation, and global dispersal (migration). The UNTB has two key parameters (elements): i) the immigration rate (*M*_i_) that controls the coupling of a local community to the metacommunity; ii) the speciation rate (also known as the fundamental biodiversity number *θ*) that can be interpreted as the rate at which new individuals are added to the metacommunity due to speciation. The UNTB assumes that the species abundance distribution of each community sample can be described by the multinomial (MN) distribution, which is parameterized by the previously mentioned two parameters. Testing the UNTB model is then computationally equivalent to testing the goodness‐of‐fitting to the MN distribution. In this study, Harris et al. Bayesian fitting framework was adopted for testing the UNTB by approximating the neutral models with the HDP.^[^ ^8^ ^]^ The advantage of Harris et al. approach is that it can simultaneously and efficiently estimate the migration rates among multiple sites, hence, it was termed as HDP‐MSN model.^[^ ^8^ ^]^

Assuming a potentially infinite number of species can be observed in the local community, the stationary distribution of observing local population *i* can be modeled with a Dirichlet process (DP), i.e.,

(4) $$\begin{array}{c} \left(\bar{\pi_{i}}\right|M_{i},\bar{\beta }∼\mathrm{DP}\left(M_{i},\bar{\beta }\right) \end{array}$$

where$\bar{\beta }\left(\beta_{1},\ldots ,\beta_{S}\right)$ is the relative frequency of each species in the metacommunity.

At the metacommunity level, a Dirichlet process is also applicable and the metacommunity distribution can be modeled with a stick breaking process, i.e.,

(5) $$\begin{array}{c} \bar{\beta }∼\mathrm{Stick}\left(\theta \right) \end{array}$$

Given that both local community and metacommunity follow Dirichlet processes, the problem can be formulated as a HDP in the domain of machine learning.^[^ ^8^, ^39^, ^40^ ^]^

Furthermore, DP can be formulated as the so‐called Chinese restaurant process, from which Antoniak equation can be derived.^[^ ^41^ ^]^ The Antoniak equation represents for the number of types (or species) (*S*) observed following *N* draws from a Dirichlet process with concentration parameter *θ* and is with the following form

(6) $$\begin{array}{c} P\left(S\left|\theta ,N\right)\right)=s\left(N,S\right)\theta^{S}\frac{\Gamma \left(\theta \right)}{\Gamma \left(\theta +N\right)} \end{array}$$

where *s*(*N*, *S*) is the unsigned Stirling number of the first kind and Γ(.) is the gamma function.

By combining Equations (4)–(6) and the previously mentioned multinominal (MN) distribution of the community samples, Harris et al. obtained their full HDP‐MSN model.^[^ ^8^ ^]^ They further developed an efficient Gibbs sampler for the UNTB‐HDP approximation, which is a type of Bayesian Markov Chain Monte Carlo algorithm.

By treating the coronavirus infections at different sites (e.g., provinces of China or different countries/regions of the world, in this study) as a “metacommunity” consisting of *N* local communities (e.g., each local community corresponding to a province), the above‐described metacommunity model can be built with the dataset of daily incremental infections, nationally or internationally. Different from traditional metacommunity concept, here the “metacommunity” is actually a metapopulation consisting of *N* local populations. However, if the local infections are treated at a particular time point (e.g., day) as a local sub‐population, then the virus sub‐populations at different time points can be considered as a total population (or “species” in the terminology of community ecology). With this conceptual transformation, the concept and models for metacommunity and Hubbell's UNTB can be readily applied to the metapopulation of coronavirus infections without a need to revise the models. With this translational scheme, the fundamental biodiversity number (speciation rate: *θ*) from the previously introduced HDP‐MSN model can be used to approximate the average local (contagion) infection rate. Similarly, the fundamental migration (dispersal) number (*M*) can be used to approximate the average infection rate through migration.

### PLEC‐ITR Model for Predicting Inflection Points of COVID‐19 Outbreaks

A hallmark of coronaviruses outbreaks seems to be exponential or near exponential growth of infections, and the peak time and amplitude or the outbreak inflection points of the virus are of obvious importance both theoretically and practically. The PLEC model was adopted for estimating the inflection (turning) points of coronavirus infections. The PLEC model is of the following form

(7) $$\begin{array}{c} I=cT^{w}\exp\left(dT\right) \end{array}$$

Ma introduced it for general DAR,^[^ ^9^, ^10^, ^36^ ^]^ DTR, and diversity–time–area relationship. Another extension to the model was the derivation of two parameters by Ma:^[^ ^9^, ^10^, ^36^, ^42^ ^]^ the maximal accrual diversity and corresponding time or space points. The maximal accrual diversity can be used to estimate the so‐termed potential diversity (also known as “dark” diversity), which takes into accounts of the species that are absent locally (in local communities) but present regionally (in metacommunities).^[^ ^10^ ^]^

With Equation (7), *T* is the time or days (starting from a specified date), and *I* is the number of cumulative infections, *c, d*, and *w* are three parameters to be estimated from the observed data. Ma derived the maximum of Equation (7) by solving the following equation^[^ ^36^ ^]^

(8) $$\begin{array}{c} \frac{df(I)}{dT}={\left[cT^{w}\exp\left(dT\right)\right]}^{\prime }=0 \end{array}$$

which leads to

(9) $$\begin{array}{c} I_{\max}=c{\left(-\frac{w}{d}\right)}^{w}\exp\left(-w\right)=cT_{\max}^{w}\exp\left(-w\right) \end{array}$$

(10) $$\begin{array}{c} T_{\max}=-\frac{w}{d}\left(w>0,d<0\right) \end{array}$$

Theoretically, Equations (9) and (10) can be utilized to estimate the *maximal infection number* (*I*_max_) and corresponding *inflection time* point (*T*_max_) at which *I*_max_ occurs.

Parameter *d* is of particular interests: *exp*(d*T*) in Equation (7) is the exponential decay term that acts as taper‐off parameter to eventually overwhelm the power law behavior (exponential or near exponential growth) at very large value of *T*. For Equation (*7*) to achieve maximum of realistic biomedical meaning (*T*_max_ > 0), (*w* > 0 and *d* < 0) are necessary conditions.

Since the taper‐off effects of parameter *d* is usually rather weak before the infection peaks, it is reasonable to consider *w* as an approximation of the *infection growth rate* and *c* as an approximation of the *initial infection number*. To fit PLEC model (Equation (7)), nonlinear optimization method was used, specifically the R function “nlsLM” or R package “minpack.lm” (https://www.rdocumentation.org/packages/minpack.lm/versions/1.2-1/topics/nlsLM).^[^ ^43^ ^]^ Since *T*_max_ > 0 is a necessary condition for the PLEC model to be biomedically sound, a constraint *d* < 0 was added for the nonlinear fitting of the PLEC model.

It is particularly worthy of noticing that the dependent variable (*I*) of PLEC, almost unavoidably, declines after the inflection point (maximum) due to the taper‐off parameter (*d*). Nevertheless, this should not be an issue in the case of this study, since the purpose is to detect the inflection point (*T*_max_) as well as the corresponding maximum (*I*_max_). In other words, the declining piece of PLEC curve after the inflection point is “irrelevant” for the purpose, since both *T*_max_ and *I*_max_ refer to the *first passage time* or the *first passage number* of maximal infections. Obviously, the actually observed cumulative numbers of infections after the inflection point usually still increase, but it is irrelevant to the prediction of the inflection point.

### Fisher Information Based Approach for Detecting Tipping Points

Fisher information was developed by Fisher as a measure of the amount of information about a particular parameter (or system characteristic) that can be obtained by observation,^[^ ^44^ ^]^ namely, the probability of observing various conditions (*p*(*s*)) of the system

(11) $$\begin{array}{c} I=\int \frac{ds}{p(s)}{\left[\frac{dp(s)}{ds}\right]}^{2} \end{array}$$

where *s* is the condition of state (*s*) of a system. Sundstrom et al. developed a framework for detecting tipping points based on Fisher information,^[^ ^45^ ^]^ which was used for detecting the tipping points associated with COVID‐19 outbreaks. In Sundstrom et al. framework,^[^ ^45^ ^]^ FI is computed from the probability density function [*p*(*n*)]

(12) $$\begin{array}{c} \mathrm{FI}=4\sum_{n=1}^{N}{\left[q_{n}-q_{n+1}\right]}^{2} \end{array}$$

where *N* is the number of states in one time window and $q_{n}=\sqrt{p(n)}$. Intuitively, the above formula outputs the FI value based on the time points within each states. For example, if all time points belong to the same state, FI = 4, which is the maximum and suggests no TP. If no adjacent time points belong to the same state, FI = 0, which corresponds to a new state, potentially a TP. In general, the minimum of FI may correspond to TP.

### Statistical Analysis

*Preprocessing of data*: None. *Data presentation*: Numbers of infections are in the form of natural numbers (0, 1, 2, ……). *Sample size (n) for each statistical analysis*: See Tables S1 and S2 in the Supporting Information. *Statistical methods used to assess significant differences*: Standard permutation (randomization tests)^[^ ^46^ ^]^ was used with *p*‐value = 0.05. *Software used for statistical* analysis: Described in previous sub‐sections in the Experimental Section.

## Data Availability

All the datasets are available in the public domain: https://news.qq.com/zt2020/page/feiyan.htm#/ or https://news.ifeng.com/c/special/7tPlDSzDgVk for the (confirmed) infections in China; for the infection datasets outside China: https://github.com/CSSEGISandData/COVID-19, managed by Johns Hopkins University. The SARS infection datasets were from the WHO (https://www.who.int/csr/sars/country/en/).

## Conflict of Interest

The author declares no conflict of interest.

## Supporting information

Supporting Information
