# Supplementary material for: Predicting the Outbreak Risks and Inflection Points of COVID‐19 Pandemic with Classic Ecological Theories
Source: Adv Sci (Weinh). 2020 Sep 24;7(21):2001530. doi: 10.1002/advs.202001530 (PMC7536942; doi:10.1002/advs.202001530)
Supplement: Supplementary file 1 — Supporting Information [file ADVS-7-2001530-s001.pdf]

© 2020 Wiley-VCH GmbH

ADVANCED SCIENCE
Open Access

Supporting Information

for *Adv. Sci.*, DOI: 10.1002/advs.202001530

Predicting the Outbreak Risks and Inflection Points of COVID-19 Pandemic with Classic Ecological Theories

Zhanshan (Sam) Ma

Online Supplementary Tables & Figures for: Ma ZS (2020) Predicting the outbreak risks and inflection points of COVID-19 pandemic with classic ecological theories. *Advanced Science*.

Table S1. The spatiotemporal TPL (Taylor's power law) parameters fitted with the primary fitting scheme to the daily cumulative infections and daily incremental infections of COVID-19 and SARS infections, respectively (*see* Table S2 for the results of the alternative fitting scheme)

**Datasets**		**Log-Liner Regression (LLR)**					***Geometric Mean Regression (GMR)**			N_{Time} ***	N_{Space} ***
		b	**ln(*a*)**	M_0	***R***	*******P*-value**	***b***	**ln(*a*)**	M_0		
**COVID-19**											
Daily Cumulative Infections of COVID-19	New York, USA	2.083	-1.418	3.703	0.995	0.000	2.093	-1.471	3.839	103	59
	New Jersey, USA	1.935	-0.170	1.199	0.992	0.000	1.952	-0.299	1.369	100	22
	California, USA	2.250	-1.918	4.635	0.991	0.000	2.270	-2.009	4.862	139	56
	Illinois, USA	2.297	-2.147	5.237	0.989	0.000	2.322	-2.232	5.412	141	98
	Massachusetts, USA	2.204	-2.203	6.235	0.998	0.000	2.207	-2.227	6.330	133	14
	USA	2.398	-3.987	17.331	0.972	0.000	2.468	-4.571	22.517	143	57
	Hubei, China	2.254	-4.347	31.999	0.998	0.000	2.260	-4.384	32.452	154	17
	China	2.191	-3.356	16.751	0.982	0.000	2.231	-3.580	18.325	154	34
	#Hubei, China (Apr.10)	2.259	-3.709	19.030	0.997	0.000	2.267	-3.759	19.446	91	17
	#China (April 10)	2.153	-2.599	9.525	0.980	0.000	2.198	-2.838	10.692	91	34
	World	2.165	-1.562	3.820	0.988	0.000	2.192	-1.740	4.309	143	187
Mean		2.199	-2.492	10.860	0.989	0.000	2.224	-2.646	11.778	126.545	54.091
Standard Error		0.036	0.380	2.825	0.003	0.000	0.039	0.397	3.001	7.532	15.322
**SARS**											
Cumulative of SARS	World	2.020	-2.293	9.475	0.965	0.000	2.094	-2.452	9.408	136	23
	World except for China	1.902	-2.112	10.409	0.931	0.000	2.042	-2.372	9.744	136	22
**COVID-19**											
Daily Incremental Infections of COVID-19	New York, USA	1.747	1.072	0.238	0.986	0.000	1.771	1.037	0.261	102	59
	New Jersey, USA	2.114	-0.632	1.765	0.980	0.000	2.157	-0.804	2.005	99	22
	California, USA	1.632	1.475	0.097	0.986	0.000	1.655	1.452	0.109	138	56
	Illinois, USA	1.541	1.251	0.099	0.982	0.000	1.570	1.257	0.110	140	97
	Massachusetts, USA	1.823	0.617	0.473	0.981	0.000	1.859	0.490	0.566	132	14
	USA	1.645	1.532	0.093	0.983	0.000	1.674	1.400	0.125	142	57
	Hubei, China	1.901	2.348	0.074	0.993	0.000	1.915	2.322	0.079	153	17
	China	1.781	1.978	0.079	0.983	0.000	1.811	1.953	0.090	153	34
	#Hubei, China (Apr 10)	1.904	1.707	0.152	0.991	0.000	1.922	1.663	0.165	90	17
	#China (April 10)	1.781	1.435	0.159	0.982	0.000	1.814	1.391	0.181	90	34
	World	1.719	1.688	0.096	0.986	0.000	1.744	1.609	0.115	142	187
Mean		1.781	1.316	0.302	0.985	0.000	1.808	1.252	0.346	126	54
Standard Error		0.047	0.238	0.150	0.001	0.000	0.048	0.250	0.171	7.532	15.296
**SARS**											
Daily Incremental of SARS	World	1.546	2.829	0.006	0.906	0.000	1.707	3.148	0.012	136	18
	World except for China	1.537	2.806	0.005	0.815	0.000	1.887	3.630	0.017	136	17

*: Given the minor differences between LLR and GMR approaches, plus $N>15$, we adopted the results from LLR.

**: *P*-value<*0.001* indicated that all the model fittings are statistically significant; *R* (linear correlation coefficient) further confirmed the exceptional significance level of the TPL fittings.
***: N_{Time} time points are used to compute *V-M* pairs. N_{Space} points are regressed to build the spatiotemporal TPL model (see Table S2 for the results from using the alternative scheme to build the TPL model).
#: Starting dates: January 11th for China, January 22nd for USA and whole world; Ending dates: all datasets end on June 12th except for the two Chinese datasets ended on April 10th as noted.

Table S2. The alternative (secondary) spatiotemporal TPL parameters fitted to the datasets of COVID-19 infections *via* regression of *V-M* (variance-mean) pairs across time series points*,#

Datasets		Log-Liner Regression (LLR)					N_{Time}	N_{Space}
		b	**ln(*a*)**	M_0	***R***	****P*-value**		
**COVID-19**								
Cumulative	New York, USA	2.302	0.447	0.710	0.997	0.000	101	58
	New Jersey, USA	1.940	0.490	0.593	0.999	0.000	97	22
	California, USA	2.587	-1.527	2.617	0.997	0.000	131	57
	Illinois, USA	2.449	0.910	0.534	0.996	0.000	95	103
	Massachusetts, USA	2.037	-0.240	1.260	0.998	0.000	99	14
	USA	2.161	0.160	0.871	0.996	0.000	139	57
	Hubei, China	2.255	0.041	0.968	0.997	0.000	144	18
	China	2.205	1.503	0.287	0.997	0.000	146	34
	Hubei, China (April 10)	2.222	0.267	0.805	0.996	0.000	79	17
	China (April 10)	2.210	1.476	0.295	0.997	0.000	83	34
	World	1.975	3.364	0.032	0.992	0.000	143	187
**Mean**		2.213	0.626	0.816	0.997	0.000	114	55
**Standard Error**		0.058	0.372	0.208	0.001	0.000	7.925	15.436
Daily Incremental	New York, USA	2.406	1.086	0.462	0.979	0.000	101	58
	New Jersey, USA	1.855	0.709	0.436	0.979	0.000	95	22
	California, USA	2.248	1.630	0.271	0.990	0.000	107	57
	Illinois, USA	2.885	0.984	0.593	0.961	0.000	93	103
	Massachusetts, USA	2.094	-0.338	1.362	0.986	0.000	96	14
	USA	1.962	1.470	0.217	0.989	0.000	111	57
	Hubei, China	1.859	2.562	0.051	0.988	0.000	63	18
	China	2.037	2.545	0.086	0.989	0.000	138	34
	Hubei, China (April 10)	1.857	2.607	0.048	0.987	0.000	56	17
	China (April 10)	2.217	2.076	0.181	0.984	0.000	82	34
	World	2.157	2.147	0.156	0.993	0.000	142	187
**Mean**		2.143	1.589	0.351	0.984	0.000	99	55
**Standard Error**		0.092	0.279	0.115	0.003	0.000	8.020	15.436

*: The alternative (secondary) scheme to fit the TPL model: N_{Space} spatial points are used to compute *V-M* (variance-mean) pairs; N_{time} time points are regressed to build the TPL model.
**: *P*-value<*0.001* indicated that all the model fittings are statistically significant; *R* (linear correlation coefficient) further confirmed the exceptional significance level of the TPL fittings.
#: The starting and ending dates of the data collections are exactly the same as in Table S1.

Table S3. The randomization (permutation) test results (the *P*-values) of the TPL parameters between the *primary* and *alternative* (secondary) fitting schemes (*see* Table S1 & S2 for the TPL parameters fitted with the primary scheme and alternative scheme, respectively) (*P*-value<0.05 suggesting significant difference between the two schemes in the respective TPL parameters)*

Primary Fitting Scheme	Alternative Fitting Scheme	TPL Parameters	Cumulative Infections	Incremental Infections
New York	New York	b	0.070	0.000
		$\ln(a)$	0.022	0.983
		M_0	0.074	0.140
New Jersey	New Jersey	b	0.936	0.130
		$\ln(a)$	0.077	0.054
		M_0	0.034	0.052
California	California	b	0.000	0.000
		$\ln(a)$	0.244	0.472
		M_0	0.000	0.007
Illinois	Illinois	b	0.244	0.000
		$\ln(a)$	0.000	0.195
		M_0	0.000	0.000
Massachusetts	Massachusetts	b	0.028	0.130
		$\ln(a)$	0.002	0.202
		M_0	0.002	0.226
USA	USA	b	0.000	0.000
		$\ln(a)$	0.000	0.912
		M_0	0.000	0.379
Hubei	Hubei	b	0.999	0.453
		$\ln(a)$	0.373	0.159
		M_0	0.233	0.113
China	China	b	0.975	0.004
		$\ln(a)$	0.138	0.000
		M_0	0.158	0.792
World	World	b	0.018	0.000
		$\ln(a)$	0.000	0.084
		M_0	0.005	0.412
Percentages with Significant Differences (%)		b	44.4	66.7
		$\ln(a)$	55.6	11.1
		M_0	66.7	22.2

*Theoretically, the TPL model can be fitted with either primary or secondary fitting scheme, to either cumulative or daily incremental infections dataset, as demonstrated previously. Practically, given that the scaling parameter (b) is more stable (also see Table S4 for the comparisons of different regions) with the primary fitting scheme to the cumulative infection numbers, and more importantly that the infection critical threshold (M_0) derived from the primary scheme fitted to the cumulative infection numbers is more convenient to compare with practical observations, we decided to use the primary fitting scheme fitted to the cumulative infection numbers as the primary results, which are the main grounds for the inferences and discussion in the main manuscript.

** Note that the randomization tests were designed to test the differences of the TPL parameters of the same dataset (New York *vs*. New York) fitted with different fitting schemes (*e.g.,* Primary *vs*. Secondary). In contrast, the randomization tests in Table S4 below were designed to detect the different between different locations (*e.g.*, New York *vs*. New Jersey).

Table S4. The randomization (permutation) test results (*P*-values) of the TPL (Taylor's power law) parameters for the COVID-19 from different regions (with significance level *P*-value set to *P*=0.05) (see Table S1-S2 for the TPL parameters)*

Treatment A	Treatment B	TPL Parameter	Primary Fitting Scheme		Alternative Fitting Scheme	
			Cumulative	Incremental	Cumulative	Incremental
New York	New Jersey	b	0.003	0.000	0.000	0.000
		$\ln(a)$	0.001	0.000	0.913	0.450
		M_0	0.004	0.000	0.601	0.884
New York	California	b	0.019	0.079	0.000	0.018
		$\ln(a)$	0.222	0.002	0.000	0.041
		M_0	0.415	0.005	0.000	0.023
New York	Illinois	b	0.002	0.004	0.040	0.000
		$\ln(a)$	0.021	0.076	0.365	0.852
		M_0	0.092	0.000	0.139	0.151
New York	Massachusetts	b	0.051	0.502	0.000	0.080
		$\ln(a)$	0.044	0.062	0.188	0.036
		M_0	0.018	0.050	0.225	0.003
New York	USA	b	0.000	0.099	0.001	0.000
		$\ln(a)$	0.000	0.068	0.323	0.282
		M_0	0.000	0.145	0.374	0.000
New York	Hubei	b	0.137	0.248	0.412	0.000
		$\ln(a)$	0.000	0.000	0.375	0.000
		M_0	0.000	0.113	0.341	0.000
New York	China	b	0.426	0.781	0.156	0.000
		$\ln(a)$	0.000	0.000	0.042	0.000
		M_0	0.000	0.033	0.063	0.000
New York	World	b	0.108	0.558	0.000	0.000
		$\ln(a)$	0.698	0.002	0.000	0.002
		M_0	0.909	0.010	0.000	0.001
New Jersey	California	b	0.000	0.000	0.000	0.000
		$\ln(a)$	0.003	0.000	0.000	0.000
		M_0	0.032	0.000	0.000	0.000
New Jersey	Illinois	b	0.000	0.000	0.000	0.000
		$\ln(a)$	0.000	0.000	0.641	0.762
		M_0	0.000	0.000	0.056	0.013
New Jersey	Massachusetts	b	0.000	0.035	0.006	0.038
		$\ln(a)$	0.003	0.021	0.015	0.035
		M_0	0.000	0.036	0.011	0.037
New Jersey	USA	b	0.162	0.000	0.000	0.104
		$\ln(a)$	0.207	0.000	0.177	0.025
		M_0	0.322	0.000	0.187	0.231
New Jersey	Hubei	b	0.103	0.327	0.000	0.939
		$\ln(a)$	0.005	0.000	0.254	0.000
		M_0	0.022	0.000	0.118	0.000

New Jersey	China	b	0.042	0.001	0.027	0.000
		ln(a)	0.000	0.000	0.224	0.000
		M_0	0.020	0.000	0.095	0.000
New Jersey	World	b	0.010	0.000	0.551	0.042
		ln(a)	0.057	0.000	0.000	0.067
		M_0	0.178	0.000	0.069	0.721
California	Illinois	b	0.465	0.125	0.023	0.000
		ln(a)	0.487	0.029	0.000	0.021
		M_0	0.520	0.926	0.000	0.000
California	Massachusetts	b	0.652	0.018	0.000	0.094
		ln(a)	0.681	0.001	0.000	0.000
		M_0	0.418	0.001	0.000	0.000
California	USA	b	0.036	0.808	0.000	0.000
		ln(a)	0.000	0.793	0.000	0.455
		M_0	0.000	0.942	0.000	0.128
California	Hubei	b	0.985	0.023	0.000	0.000
		ln(a)	0.002	0.000	0.001	0.000
		M_0	0.000	0.647	0.001	0.000
California	China	b	0.669	0.175	0.000	0.000
		ln(a)	0.049	0.000	0.000	0.000
		M_0	0.000	0.704	0.000	0.000
California	World	b	0.125	0.043	0.000	0.016
		ln(a)	0.358	0.184	0.000	0.002
		M_0	0.445	0.977	0.000	0.020
Illinois	Massachusetts	b	0.373	0.005	0.001	0.004
		ln(a)	0.916	0.000	0.195	0.203
		M_0	0.519	0.000	0.000	0.011
Illinois	USA	b	0.028	0.009	0.000	0.000
		ln(a)	0.000	0.034	0.085	0.307
		M_0	0.000	0.880	0.018	0.000
Illinois	Hubei	b	0.780	0.013	0.042	0.000
		ln(a)	0.000	0.000	0.246	0.000
		M_0	0.000	0.625	0.000	0.000
Illinois	China	b	0.394	0.025	0.001	0.000
		ln(a)	0.013	0.000	0.261	0.000
		M_0	0.000	0.652	0.002	0.000
Illinois	World	b	0.001	0.000	0.000	0.000
		ln(a)	0.027	0.000	0.000	0.000
		M_0	0.065	0.904	0.000	0.000
Massachusetts	USA	b	0.525	0.389	0.000	0.097
		ln(a)	0.499	0.380	0.129	0.000
		M_0	0.443	0.279	0.130	0.000
Massachusetts	Hubei	b	0.853	0.770	0.001	0.004
		ln(a)	0.268	0.009	0.573	0.000

		M_0	0.152	0.000	0.610	0.000
Massachusetts	China	b	0.945	0.804	0.161	0.292
		ln(a)	0.328	0.000	0.061	0.000
		M_0	0.225	0.000	0.035	0.000
Massachusetts	World	b	0.714	0.276	0.315	0.569
		ln(a)	0.435	0.016	0.000	0.000
		M_0	0.297	0.024	0.024	0.061
USA	Hubei	b	0.610	0.015	0.012	0.014
		ln(a)	0.889	0.114	0.681	0.000
		M_0	0.595	0.567	0.636	0.000
USA	China	b	0.110	0.028	0.407	0.000
		ln(a)	0.673	0.062	0.000	0.000
		M_0	0.979	0.648	0.000	0.000
USA	World	b	0.000	0.157	0.000	0.000
		ln(a)	0.000	0.459	0.000	0.019
		M_0	0.000	0.932	0.000	0.349
Hubei	China	b	0.782	0.717	0.523	0.000
		ln(a)	0.489	0.421	0.018	0.959
		M_0	0.140	0.970	0.000	0.000
Hubei	World	b	0.426	0.043	0.000	0.000
		ln(a)	0.000	0.043	0.000	0.082
		M_0	0.000	0.656	0.000	0.011
China	World	b	0.767	0.305	0.000	0.000
		ln(a)	0.004	0.150	0.000	0.000
		M_0	0.000	0.679	0.000	0.000
Percentages with Significant Differences (%)		b	36.1	52.8	80.6	80.6
		ln(a)	58.3	69.4	52.8	72.2
		M_0	52.8	50.0	61.1	80.6

*As noted previously, the randomization tests here were designed to detect the differences in TPL parameters between difference locations, rather than different fitting approaches as in the previous Table S3. The results here show that the TPL scaling parameter (b) is still much more stable (less differences) with the primary fitting scheme fitted to cumulative infection numbers. Both the results here and previous randomization tests support our choice of the “primary fitting scheme to the cumulative infections” as the primary approach (results) in this study.

Table S5. The randomization (permutation) test results (*P*-values) for the TPL (Taylor's power law) parameters between COVID-19 and SARS (fitted with the primary fitting scheme only)*

**Treatment A** (COVID-19)	**Treatment B** (SARS-World)	**TPL Parameters**	***P*-value**	
			**Cumulative**	**Incremental**
New York	SARS	b	0.395	0.168
		ln(a)	0.043	0.000
		M_0	0.000	0.003
New Jersey	SARS	b	0.204	0.000
		ln(a)	0.000	0.000
		M_0	0.000	0.000
California	SARS	b	0.000	0.432
		ln(a)	0.402	0.000
		M_0	0.001	0.013
Illinois	SARS	b	0.012	0.970
		ln(a)	0.717	0.000
		M_0	0.001	0.025
Massachusetts	SARS	b	0.010	0.003
		ln(a)	0.868	0.000
		M_0	0.269	0.000
USA	SARS	b	0.000	0.273
		ln(a)	0.039	0.004
		M_0	0.109	0.011
Hubei	SARS	b	0.001	0.001
		ln(a)	0.000	0.269
		M_0	0.000	0.000
China	SARS	b	0.092	0.164
		ln(a)	0.056	0.000
		M_0	0.051	0.003
World	SARS	b	0.049	0.063
		ln(a)	0.201	0.005
		M_0	0.007	0.070
Percentages with Significant Differences (%)		b	66.7	33.3
		ln(a)	44.4	88.9
		M_0	66.7	88.9

*The test shows that COVID-19 and SARS are significantly different in their TPL parameters in a majority of the comparisons, particularly in "COVID-19 world *vs*. SARS world" comparison. Obviously, the most meaningful comparison is the last one (COVID-19 world *vs*. SARS world), which suggests the significant difference between COVID-19 and SARS in the scaling parameter b and infection critical threshold M_0 for the cumulative infections, which were the primary approach/datasets.

Table S6. The metapopulation model parameters fitted to the daily increments of COVID-19 and SARS infections, respectively, by adapting Hubbell's UNTB (unified neutral theory of biodiversity) implemented with Harris *et al*. (2017) multi-site neutral (MSN) model for the metacommunity*,**

Datasets	*M/θ*	*M*	*θ*	Meta-population			Local Population		
				N_M	*N*	P_M	N_L	*N*	P_L
**COVID-19**									
New York	7.659	112.022	14.626	2500	2500	1.000	1027	2500	0.411
New Jersey	26.022	335.001	12.874	2500	2500	1.000	2252	2500	0.901
California	3.812	63.979	16.782	2500	2500	1.000	1433	2500	0.573
Illinois	7.489	103.278	13.792	2499	2500	0.999	939	2500	0.376
Massachusetts	44.365	572.599	12.907	2500	2500	1.000	939	2500	0.376
USA	18.192	237.288	13.043	2498	2498	1.000	1254	2498	0.502
Hubei (April 10)	6.840	74.363	10.872	1548	2500	0.619	1701	2500	0.680
Hubei (June 12)	6.079	72.695	11.958	1398	2500	0.559	1688	2500	0.675
China (April 10)	6.279	91.408	14.557	2454	2496	0.983	2247	2496	0.900
China (June 12)	2.677	76.944	28.743	1645	2498	0.659	1499	2498	0.600
World	4.936	83.360	16.889	2496	2497	0.999	2381	2497	0.954
Mean	12.214	165.722	15.186	2230.727	2499	0.893	1578.2	2499	0.632
Standard Error	3.837	48.020	1.465	136.724	0.447	0.055	160.294	0.447	0.064
**SARS**									
World	0.6	16.241	27.169	2439	2494	0.978	2148	2494	0.861
World-except China	0.5	15.094	32.219	2363	2495	0.947	2144	2495	0.859

*θ: Local contagion rate; *M*: Mean migration rate; *m*: immigration probability; *N*=2500 is the number of Gibb samples selected from 25000 simulated communities performed to test the fitting of Harris *et al*. (2015) HDP-MSN model; N_M & N_L are the number of simulations that passed the neutrality test (by comparing with actual daily incremental infection numbers), respectively. P_M and P_L are the *pseudo-P* values from performing the neutrality test at meta-population and local population levels, respectively. When *pseudo-P* value>*0.05*, it indicates that the dataset satisfied the neutral model, and the parameters (θ & *M*), especially $M/\boldsymbol{\theta}$ can be harnessed to assess and interpret the relative importance of local contagion vs. migration in spreading the coronaviruses infections.

** The durations of data collections were the same as in Table S1.

Table S7. The PLEC-ITR (Power Law with Exponential Cutoff for describing the Infection Time Relationship) model, fitted with nonlinear optimization for daily cumulative COVID-19 infections in China*

Country or Province	City or Province	*z*	*P(z)*	*d*	*P(d)*	*c*	*P(c)*	R^2	T_{max}	I_{max}
Hubei Province	Wuhan	9.006	0.000	-0.188	0.000	0.000	0.638	0.986	48	49193.4
	Xiaogan	2.856	0.000	-0.091	0.000	3.232	0.001	0.993	32	3534.6
	Huanggang	3.007	0.000	-0.094	0.000	1.806	0.000	0.994	32	2979.4
	Jingzhou	3.288	0.000	-0.102	0.000	0.468	0.005	0.993	32	1607.0
	Ezhou	3.544	0.000	-0.110	0.000	0.221	0.024	0.992	32	1413.2
	Suizhou	2.340	0.000	-0.081	0.000	5.357	0.000	0.992	29	1336.7
	Xiangyang	1.876	0.000	-0.070	0.000	16.522	0.000	0.992	27	1210.0
	Huangshi	2.268	0.000	-0.081	0.000	5.248	0.000	0.993	28	1033.6
	Yichang	2.476	0.000	-0.083	0.000	2.480	0.000	0.993	30	946.2
	Jingmen	2.558	0.000	-0.079	0.000	1.630	0.019	0.981	32	929.5
	Xianning	1.933	0.000	-0.059	0.000	6.804	0.027	0.964	33	849.6
	Shiyan	2.247	0.000	-0.068	0.000	2.472	0.000	0.999	33	673.2
	Xiantao	3.034	0.000	-0.097	0.000	0.350	0.000	0.997	31	584.6
	Tianmen	3.963	0.000	-0.125	0.000	0.030	0.164	0.982	32	508.0
	Enshi	1.701	0.000	-0.053	0.000	3.851	0.000	0.993	32	254.6
	Qianjiang	2.650	0.000	-0.074	0.000	0.218	0.178	0.968	36	204.4
	Shennongjia	0.618	0.000	-0.026	0.000	2.809	0.000	0.897	24	10.8
China	Hubei	8.440	0.000	-0.188	0.000	0.000	0.389	0.994	45	66767.0
	Guangdong	2.988	0.000	-0.091	0.000	0.800	0.001	0.993	33	1386.8
	Henan	2.657	0.000	-0.085	0.000	1.960	0.000	0.996	31	1306.9
	Zhejiang	2.102	0.000	-0.070	0.000	8.069	0.000	0.986	30	1239.5
	Hunan	2.452	0.000	-0.080	0.000	2.732	0.000	0.995	31	1049.2
	Anhui	2.637	0.000	-0.086	0.000	1.720	0.000	0.996	31	1019.6
	Jiangxi	3.001	0.000	-0.097	0.000	0.653	0.000	0.995	31	970.8
	Shandong	1.805	0.000	-0.033	0.000	3.731	0.024	0.981	55	853.7
	Jiangsu	2.510	0.000	-0.080	0.000	1.385	0.000	0.999	31	646.3
	Chongqing	1.946	0.000	-0.058	0.000	4.343	0.000	0.998	34	579.5
	Sichuan	2.061	0.000	-0.058	0.000	2.660	0.000	0.999	36	534.9
	Heilongjiang	3.490	0.000	-0.108	0.000	0.088	0.000	0.997	32	491.2
	Beijing	2.257	0.000	-0.066	0.000	1.354	0.000	0.996	34	408.5
	Shanghai	2.320	0.000	-0.073	0.000	1.144	0.000	0.994	32	344.9
	Hebei	2.559	0.000	-0.076	0.000	0.505	0.000	0.996	34	318.7
	Fujian	1.805	0.000	-0.060	0.000	3.929	0.000	0.994	30	300.8
	Guangxi	1.773	0.000	-0.053	0.000	2.984	0.000	0.998	33	252.9
	Shaanxi	1.848	0.000	-0.063	0.000	3.075	0.000	0.995	29	250.1
	Yunnan	1.735	0.000	-0.056	0.000	2.625	0.000	0.988	31	176.3
	Hainan	2.284	0.000	-0.068	0.000	0.554	0.000	0.993	33	170.9
	Guizhou	3.632	0.000	-0.118	0.000	0.023	0.004	0.994	31	152.6
	Tianjin	2.642	0.000	-0.075	0.000	0.158	0.000	0.997	35	136.6
	Shanxi	2.260	0.000	-0.075	0.000	0.598	0.000	0.994	30	136.4
	Liaoning	1.759	0.000	-0.058	0.000	1.784	0.000	0.997	30	123.9
	HongKong	2.025	0.000	-0.025	0.011	0.162	0.069	0.987	80	151.2

	Jilin	2.978	0.000	-0.099	0.000	0.074	0.023	0.983	30	95.7
	Gansu	1.857	0.000	-0.064	0.000	1.159	0.000	0.994	29	93.7
	Xinjiang	2.583	0.000	-0.078	0.000	0.123	0.000	0.993	33	77.5
	Neimenggu	1.853	0.000	-0.056	0.000	0.728	0.000	0.996	33	75.8
	Ningxia	2.235	0.000	-0.066	0.000	0.258	0.000	0.992	34	72.9
	Taiwan	NA	NA	NA	NA	NA	NA	NA	NA	NA
	Qinghai	1.163	0.000	-0.050	0.000	1.560	0.000	0.947	23	19.0
	Macao	0.963	0.000	-0.037	0.000	1.184	0.000	0.946	26	10.4
	Xizang	NA	NA	NA	NA	NA	NA	NA	NA	NA
China (Nationally)		7.419	0.000	-0.172	0.000	0.000	0.199	0.996	43	79899.1

*Data collection duration: Starting dates: January 11th for Wuhan and Hubei Province; January 13th for whole China; and January 19th for the rest of Chinese provinces. Ending dates: all datasets for China used here end at February 29th.

Table S8. #Evaluations for the *success rates* and *error rates* of the inflection (turning) point estimations based on the PLEC-ITR model (Power-Law function with Exponential Cutoff for Inflection Time Relationship), built with COVID-19 infection data in China

**Data and Regions**		***Estimated Parameters***		**Evaluation of T_{max}**					**Evaluation of I_{max}**			
**Data**	**Country /Province**	T_{max}	I_{max}	**Observed (T_{max})**	**Lower (95%)**	**Upper (95%)**	**Within 95% Interval**	**Error Rate (%)**	**Observed (Mar 6)**	**Error Rate (%)**	**Observed (Mar 12)**	**Error Rate (%)**
Hubei	Wuhan	48	49193.4	48137	46733.7	51653.1	Yes	2.1%	49797	-1.2%	49986	-1.6%
	Xiaogan	32	3534.6	3465	3357.9	3711.3	Yes	2.0%	3518	0.5%	3518	0.5%
	Huanggang	32	2979.4	2899	2830.4	3128.4	Yes	2.7%	2907	2.4%	2907	2.4%
	Jingzhou	32	1607.0	1574	1526.7	1687.4	Yes	2.1%	1580	1.7%	1580	1.7%
	Ezhou	32	1413.2	1385	1342.5	1483.9	Yes	2.0%	1394	1.4%	1394	1.4%
	Suizhou	29	1336.7	1296	1269.9	1403.5	Yes	3.0%	1307	2.2%	1307	2.2%
	Xiangyang	27	1210.0	1170	1149.5	1270.5	Yes	3.3%	1175	2.9%	1175	2.9%
	Huangshi	28	1033.6	997	981.9	1085.3	Yes	3.5%	1015	1.8%	1015	1.8%
	Yichang	30	946.2	914	898.9	993.5	Yes	3.4%	931	1.6%	931	1.6%
	Jingmen	32	929.5	918	883.0	976.0	Yes	1.2%	928	0.2%	928	0.2%
	Xianning	33	849.6	836	807.1	892.1	Yes	1.6%	836	1.6%	836	1.6%
	Shiyan	33	673.2	671	639.5	706.9	Yes	0.3%	672	0.2%	672	0.2%
	Xiantao	31	584.6	571	555.4	613.8	Yes	2.3%	575	1.6%	575	1.6%
	Tianmen	32	508.0	495	482.6	533.4	Yes	2.6%	496	2.4%	496	2.4%
	Enshi	32	254.6	251	241.9	267.3	Yes	1.4%	252	1.0%	252	1.0%
	Qianjiang	36	204.4	NA	194.2	214.6	No	NA	198	3.1%	198	3.1%
	Shennongjia	24	10.8	10	10.3	11.3	No	7.4%	11	-1.9%	11	-1.9%
	Mean	31.7	4191.5	4099.3	3982.0	4401.1		2.6%	4212.1	1.5%	4223.9	1.6%
	Std Error	1.3	3008.8	2944.4	2858.4	3159.3		0.4%	3047.4	0.2%	3059.2	0.2%
Success Rates (%)							88.2%	88.2%		100%		100%
China	Hubei	45	66767.0	65187	63428.7	70105.4	Yes	2.4%	67592	-1.2%	67786	-1.5%
	Guangdong	33	1386.8	1333	1317.5	1456.1	Yes	3.9%	1352	2.5%	1356	2.2%
	Henan	31	1306.9	1267	1241.6	1372.2	Yes	3.1%	1272	2.7%	1273	2.6%
	Zhejiang	30	1239.5	1175	1177.5	1301.5	No	5.2%	1215	2.0%	1215	2.0%
	Hunan	31	1049.2	1011	996.7	1101.7	Yes	3.6%	1018	3.0%	1018	3.0%
	Anhui	31	1019.6	989	968.6	1070.6	Yes	3.0%	990	2.9%	990	2.9%
	Jiangxi	31	970.8	934	922.3	1019.3	Yes	3.8%	935	3.7%	935	3.7%
	Shandong	55	853.7	NA	811.0	896.4	No	NA	758	11.2%	760	11.0%
	Jiangsu	31	646.3	631	614.0	678.6	Yes	2.4%	631	2.4%	631	2.4%
	Chongqing	34	579.5	575	550.5	608.5	Yes	0.8%	576	0.6%	576	0.6%
	Sichuan	36	534.9	531	508.2	561.6	Yes	0.7%	539	-0.8%	539	-0.8%
	Heilongjiang	32	491.2	480	466.6	515.8	Yes	2.3%	481	2.1%	482	1.9%
	Beijing	34	408.5	399	388.1	428.9	Yes	2.3%	422	-3.3%	436	-6.7%
	Shanghai	32	344.9	334	327.7	362.1	Yes	3.2%	342	0.8%	346	-0.3%
	Hebei	34	318.7	311	302.8	334.6	Yes	2.4%	318	0.2%	318	0.2%
	Fujian	30	300.8	293	285.8	315.8	Yes	2.6%	296	1.6%	296	1.6%
	Guangxi	33	252.9	251	240.3	265.5	Yes	0.8%	252	0.4%	252	0.4%
	Shaanxi	29	250.1	245	237.6	262.6	Yes	2.0%	245	2.0%	245	2.0%
	Yunnan	31	176.3	174	167.5	185.1	Yes	1.3%	174	1.3%	174	1.3%

	Hainan	33	170.9	168	162.4	179.4	Yes	1.7%	168	1.7%	168	1.7%
	Guizhou	31	152.6	146	145.0	160.2	Yes	4.3%	146	4.3%	146	4.3%
	Tianjin	35	136.6	135	129.8	143.4	Yes	1.2%	136	0.4%	136	0.4%
	Shanxi	30	136.4	132	129.6	143.2	Yes	3.2%	133	2.5%	133	2.5%
	Liaoning	30	123.9	121	117.7	130.1	Yes	2.3%	125	-0.9%	125	-0.9%
	HongKong	80	151.2	NA	143.6	158.8	No	NA	107	29.2%	131	13.4%
	Jilin	30	95.7	91	90.9	100.5	Yes	4.9%	93	2.8%	93	2.8%
	Gansu	29	93.7	91	89.0	98.4	Yes	2.9%	102	-8.9%	127*(1)	-35.5%
	Xinjiang	33	77.5	76	73.6	81.4	Yes	1.9%	76	1.9%	76	1.9%
	Neimenggu	33	75.8	75	72.0	79.6	Yes	1.1%	75	1.1%	75	1.1%
	Ningxia	34	72.9	71	69.3	76.5	Yes	2.6%	75	-2.9%	75	-2.9%
	Taiwan	NA	NA	NA	NA	NA	NA	NA	45	NA	49	NA
	Qinghai	23	19.0	18	18.1	20.0	No	5.3%	18	5.3%	18	5.3%
	Macao	26	10.4	10	9.9	10.9	Yes	3.8%	10	3.8%	10	3.8%
	Xizang	NA	NA	NA	NA	NA	NA	NA	1	NA	1	NA
	Mean	33.4	2560.0	2575.1	2432.0	2688.0		2.7%	2577.9	3.2%	2586.5	3.6%
	Std Error	1.7	2141.5	2125.1	2034.4	2248.6		0.2%	2168.3	0.9%	2174.5	1.2%
Success Rates (%)							85.3%	85.3%		85.3%		82.4%
China		43	79899.1	77785	75904.1	83894.1	Yes	2.6%	80718	-1.0%	81003	-1.4%

*Tibet (Xizang) had *1* case only and could not be modeled with PLEC, and is excluded from computing the rates.

The criteria for evaluating the PLEC-ITR model

We test the performance of the proposed PLEC model with two schemes. First, we evaluate the "congruency" of T_{max} (inflection time point) with the reality by comparing the *actually observed* infection number at the date of T_{max} with the estimated I_{max}. To determine the congruence, we calculate an interval [I_{max}–I_{max}5%; I_{max}+I_{max}5%] for each PLEC model. If the actually observed infection numbers falls within the interval, the estimation of T_{max} is considered as valid (or successful). It is noted that this is *not* a classic *confidence interval*, which requires the calculation of standard deviation of samples and cannot be obtained in the case of COVID-19 infections. Instead, this "homegrown" interval we computed is simply a range of I_{max} within 5% fluctuation, which we believe is a reasonable scheme for estimating the validity (success) of the T_{max} estimations.

Second, to evaluate the validity of I_{max} estimations, we used the observed infection number on the day of T_{max}, and two additional dates (March 6th and 12th for China) not used in building the PLEC model, respectively, as reference values to evaluate the performance of the PLEC model, using the following formula to calculate the error rate:

$$E(\%) = (I_{max} - I) / I_{max}\,.$$

Alternatively, the precision P(%) of the PLEC model is simply computed as the complement of E(%), *i.e.*, $P(\%)=1-E(\%)$.

As mentioned previously, we intentionally used partial datasets (Starting in January 11th for Wuhan and Hubei of China, the previous epicenter; January 13th for the whole China; and January 19th for the rest of Chinese provinces; ending dates=Feb 29th) for building the predictive PLEC models with infection datasets in China, so that the assessment of their performance in can be actually validated with practical observations. Since the outbreaks in most places of China had already peaked (passed inflection points) in the early March, and therefore, we chose March 6th and 12th as the two dates to further verify the performance of PLEC models in China, besides using the "5% interval scheme around T_{max}", as detailed above (also *see* a preprint: https://www.medrxiv.org/content/10.1101/2020.03.25.20043893v1).

Since the COVID-19 pandemic in most parts of the world apparently have not peaked yet, we could not use the "5% interval scheme" to verify predictions for the rest of the world (see Table S9 below), even if we can build successful PLEC models. For this reason, we simply use the latest observation data available for us to complete the modeling, specifically until June 12th, and do a simple test with the data on June 17th, when we finalize the results for this manuscript.

Table S9. The PLEC-ITR (Power Law with Exponential Cutoff – Infection Time Relationship) models, fitted with nonlinear optimization for daily cumulative COVID-19 infections worldwide (the cutoff or left truncation for the initial infection numbers < *100*)*

Country / State		*z*	*P(z)*	*d*	*P(d)*	*c*	*P(c)*	R^2	T_{max}	I_{max}	**Observed (June 17)**	**Error1 (%)**	**Observed (T_{max})**	**Error2 (%)**	**Start dates**
US States (33)	New Jersey	2.341	0.000	-0.028	0.000	55.204	0.000	0.998	82	161998.4	167703	-3.52	163336	-0.83	Mar 16
	California	1.942	0.000	-0.002	0.056	23.831	0.000	0.999	1138	2949580	162798	94.48	NA	NA	Mar 9
	Illinois	3.193	0.000	-0.029	0.000	1.085	0.000	0.999	109	143541.4	134185	6.52	NA	NA	Mar 17
	Massachusetts	3.630	0.000	-0.040	0.000	0.317	0.000	0.999	90	102787.3	106151	-3.27	103889	-1.07	Mar 12
	Pennsylvania	2.375	0.000	-0.025	0.000	16.941	0.000	0.999	96	81459.6	84289	-3.47	NA	NA	Mar 17
	Florida	1.405	0.000	-0.006	0.000	197.067	0.000	0.993	255	116088.4	82719	28.74	NA	NA	Mar 16
	Michigan	1.533	0.000	-0.014	0.000	224.844	0.000	0.993	110	65367.2	66497	-1.73	NA	NA	Mar 18
	Maryland	2.653	0.000	-0.019	0.000	2.416	0.000	1.000	137	78621.1	62969	19.91	NA	NA	Mar 19
	Georgia	1.935	0.000	-0.013	0.000	28.284	0.000	0.998	152	68514.9	60030	12.38	NA	NA	Mar 16
	Virginia	2.654	0.000	-0.014	0.000	1.381	0.000	0.999	185	100581.1	55775	44.55	NA	NA	Mar 19
	Louisiana	1.466	0.000	-0.013	0.000	190.056	0.000	0.982	111	43848.1	48634	-10.91	NA	NA	Mar 16
	Connecticut	2.343	0.000	-0.027	0.000	12.724	0.000	0.999	88	43977.2	45429	-3.3	NA	NA	Mar 19
	Ohio	2.229	0.000	-0.017	0.000	8.579	0.000	0.999	130	47602.2	42422	10.88	NA	NA	Mar 19
	Indiana	1.956	0.000	-0.015	0.000	22.852	0.000	1.000	135	47264	41013	13.23	NA	NA	Mar 21
	Colorado	2.294	0.000	-0.019	0.000	5.240	0.000	0.999	120	31360	29656	5.43	NA	NA	Mar 14
	Washington	1.570	0.000	-0.010	0.000	45.446	0.000	0.993	165	28683.5	26784	6.62	NA	NA	Mar 7
	Iowa	3.577	0.000	-0.036	0.000	0.060	0.001	0.998	100	24322.4	24460	-0.57	NA	NA	Mar 23
	Nebraska	3.648	0.000	-0.041	0.000	0.050	0.001	0.998	89	17050.6	17231	-1.06	NA	NA	Mar 29
	Missouri	1.171	0.000	-0.004	0.000	125.979	0.000	0.998	281	28827.5	17069	40.79	NA	NA	Mar 22
	Rhode Island	2.741	0.000	-0.033	0.000	1.302	0.000	0.999	84	15613.3	16213	-3.84	NA	NA	Mar 23
	Kansas	3.175	0.000	-0.037	0.000	0.184	0.005	0.995	86	10797.5	11644	-7.84	NA	NA	Mar 25
	Nevada	1.107	0.000	-0.001	0.062	84.168	0.000	0.997	770	43689	11854	72.87	NA	NA	Mar 20
	Delaware	2.504	0.000	-0.026	0.000	1.401	0.000	0.999	97	10681	10444	2.22	NA	NA	Mar 24
	District of Columbia	2.150	0.000	-0.019	0.000	3.507	0.000	0.999	114	10741.8	9847	8.33	NA	NA	Mar 22
	New Mexico	1.891	0.000	-0.010	0.000	5.343	0.000	0.999	188	16116.6	10065	37.55	NA	NA	Mar 25
	Oklahoma	1.063	0.000	-0.002	0.000	81.406	0.000	0.999	667	28232.8	8904	68.46	NA	NA	Mar 24
	South Dakota	1.565	0.000	-0.012	0.000	16.037	0.000	0.997	133	7052.5	6050	14.21	NA	NA	Mar 30
	Oregon	1.062	0.000	-0.002	0.036	51.454	0.000	0.997	669	17841.8	6218	65.15	NA	NA	Mar 20
	New Hampshire	1.698	0.000	-0.009	0.000	6.498	0.000	0.998	182	8155.8	5436	33.35	NA	NA	Mar 23
	North Dakota	1.850	0.000	-0.012	0.000	2.590	0.000	0.995	153	4455.8	3166	28.95	NA	NA	Mar 30
	Vermont	0.689	0.000	-0.009	0.000	102.127	0.000	0.969	76	1013.2	1130	-11.53	1075	-6.1	Mar 25
	Hawaii	0.638	0.000	-0.011	0.000	92.929	0.000	0.979	59	662.6	744	-12.28	643	2.96	Mar 27
	Montana	0.436	0.000	-0.005	0.000	112.555	0.000	0.952	93	525.7	630	-19.84	NA	NA	Mar 27
Mean		2.046	0.000	-0.018	0.001	45.673	0.000	0.994	162.5	43993.1	38822.8	17.1	NA	NA	NA
Standard Error		0.156	0.000	0.002	0.001	11.597	0.000	0.002	25.9	7813.1	7428.5	3.3	NA	NA	NA
World (78)	Italy	2.757	0.000	-0.029	0.000	13.539	0.000	0.996	94	234290.3	237828	-1.51	230555	1.59	Feb 23
	France	3.339	0.000	-0.040	0.000	2.108	0.022	0.985	83	192139.2	194805	-1.39	182354	5.09	Mar 1
	Germany	2.622	0.000	-0.031	0.000	23.260	0.001	0.988	84	185409.6	188604	-1.72	179986	2.93	Mar 1
	Iran	1.409	0.000	-0.002	0.068	310.965	0.001	0.991	582	597854.9	195051	67.37	NA	NA	Feb 26
	Turkey	2.234	0.000	-0.028	0.000	85.903	0.000	0.995	81	167127.2	182727	-9.33	170132	-1.8	Mar 19
	Saudi Arabia	4.476	0.000	-0.029	0.000	0.003	0.033	0.999	156	198822.8	141234	28.96	NA	NA	Mar 14
	Canada	2.990	0.000	-0.029	0.000	1.835	0.000	1.000	105	100462.4	101491	-1.02	NA	NA	Mar 11

China	1.148	0.000	-0.013	0.000	1628.1	0.000	0.911	89	89536.5	84903	5.17	83797	6.41	Jan 18
Belgium	2.949	0.000	-0.035	0.000	2.351	0.000	0.995	84	58810.8	60244	-2.44	57849	1.64	Mar 6
Netherlands	2.576	0.000	-0.030	0.000	6.705	0.000	0.995	85	47089.3	49412	-4.93	46328	1.62	Mar 6
Ecuador	3.161	0.000	-0.035	0.000	0.677	0.163	0.978	90	42515	48490	-14.05	NA	NA	Mar 18
United Arab Emirates	2.823	0.000	-0.016	0.000	0.586	0.000	0.999	173	72616	43364	40.28	NA	NA	Mar 18
Singapore	6.128	0.000	-0.053	0.000	0.000	0.200	0.996	115	40168.3	41216	-2.61	NA	NA	Feb 29
Portugal	1.703	0.000	-0.017	0.000	75.326	0.000	0.992	98	34023.3	37672	-10.72	NA	NA	Mar 13
Switzerland	1.934	0.000	-0.027	0.000	55.775	0.000	0.984	72	31892.9	31187	2.21	30514	4.32	Mar 5
Ukraine	2.087	0.000	-0.013	0.000	8.682	0.000	0.999	164	45305.1	33986	24.98	NA	NA	Mar 25
Poland	1.720	0.000	-0.007	0.000	22.640	0.000	0.999	246	52502.9	30701	41.53	NA	NA	Mar 14
Ireland	2.942	0.000	-0.040	0.000	1.496	0.000	0.994	74	25419.5	25341	0.31	24735	2.69	Mar 14
Romania	2.285	0.000	-0.022	0.000	4.976	0.000	0.999	105	21255.9	22760	-7.08	NA	NA	Mar 14
Israel	1.858	0.000	-0.026	0.000	40.070	0.000	0.984	72	17631.9	19783	-12.2	16712	5.22	Mar 14
Japan	6.049	0.000	-0.064	0.000	0.000	0.169	0.990	95	17346	17530	-1.06	16581	4.41	Feb 21
Austria	1.525	0.000	-0.022	0.000	117.113	0.000	0.969	71	16956.3	17203	-1.45	16242	4.21	Mar 8
Denmark	1.856	0.000	-0.020	0.000	17.356	0.000	0.997	92	12051	12494	-3.68	12201	-1.24	Mar 10
Serbia	2.428	0.000	-0.032	0.000	3.691	0.000	0.994	75	11634.7	12522	-7.63	11430	1.76	Mar 19
South-Korea	0.817	0.000	-0.010	0.000	686.092	0.000	0.955	85	11420.2	12257	-7.33	11018	3.52	Feb 20
Czech	1.342	0.000	-0.015	0.000	87.398	0.000	0.988	88	9349.1	10162	-8.69	9697	-3.72	Mar 13
Norway	1.490	0.000	-0.019	0.000	56.606	0.000	0.992	79	8506.7	8692	-2.18	8346	1.89	Mar 6
Morocco	2.283	0.000	-0.025	0.000	2.691	0.000	0.998	93	8550.6	8997	-5.22	NA	NA	Mar 22
Malaysia	1.351	0.000	-0.012	0.000	54.141	0.000	0.992	112	8228	8515	-3.49	NA	NA	Mar 9
Australia	1.427	0.000	-0.022	0.000	78.994	0.000	0.956	66	7399.8	7391	0.12	7019	5.15	Mar 10
Finland	1.951	0.000	-0.021	0.000	6.883	0.000	0.999	95	7048.5	7117	-0.97	NA	NA	Mar 13
Sudan	2.700	0.000	-0.028	0.000	0.671	0.001	0.998	95	9990	8020	19.72	NA	NA	Apr 20
Tajikistan	2.412	0.000	-0.051	0.000	5.112	0.001	0.995	47	4957.1	5221	-5.32	NA	NA	May 3
Senegal	3.162	0.000	-0.022	0.000	0.026	0.054	0.996	147	7903.6	5369	32.07	NA	NA	Mar 26
Guinea	1.828	0.000	-0.013	0.000	4.820	0.000	0.999	136	6189.1	4668	24.58	NA	NA	Apr 4
Luxembourg	0.934	0.000	-0.015	0.000	213.446	0.000	0.986	64	4057	4085	-0.69	3958	2.44	Mar 17
Hungary	1.871	0.000	-0.022	0.000	6.506	0.000	0.998	84	3983.7	4078	-2.37	4053	-1.74	Mar 21
Haiti	2.482	0.000	-0.013	0.256	0.778	0.188	0.993	192	30475.9	4688	84.62	NA	NA	May 5
El Salvador	2.274	0.000	-0.008	0.012	0.447	0.017	0.997	281	17034.5	4066	76.13	NA	NA	Apr 9
Gabon	3.408	0.000	-0.043	0.000	0.043	0.071	0.995	78	4076	4229	-3.75	NA	NA	Apr 17
Bulgaria	1.423	0.000	-0.007	0.000	9.766	0.000	0.990	201	4443.2	3542	20.28	NA	NA	Mar 20
Thailand	1.152	0.000	-0.018	0.000	83.901	0.000	0.981	63	3148.1	3135	0.42	3025	3.91	Mar 15
Greece	1.123	0.000	-0.014	0.000	63.962	0.000	0.992	83	2951.4	3203	-8.52	2937	0.49	Mar 13
Bosnia and Herzegovina	1.251	0.000	-0.011	0.000	26.108	0.000	0.997	119	2951.4	3141	-6.42	NA	NA	Mar 22
Croatia	1.138	0.000	-0.017	0.000	61.149	0.000	0.996	65	2272.8	2258	0.65	2243	1.31	Mar 19
Cuba	1.364	0.000	-0.017	0.000	21.261	0.000	0.994	80	2147.7	2280	-6.16	NA	NA	Mar 28
Maldives	0.980	0.000	-0.003	0.217	50.763	0.000	0.993	362	6125.8	2120	65.39	NA	NA	Apr 23
Estonia	1.196	0.000	-0.015	0.000	34.153	0.000	0.988	78	1879.6	1977	-5.18	1865	0.77	Mar 14
Iceland	1.203	0.000	-0.020	0.000	46.194	0.000	0.970	60	1898.2	1815	4.38	1801	5.12	Mar 12
Lithuania	0.829	0.000	-0.010	0.000	97.904	0.000	0.987	84	1685.5	1778	-5.49	NA	NA	Mar 22
Slovakia	1.785	0.000	-0.026	0.000	5.002	0.000	0.984	68	1551.8	1561	-0.59	1509	2.76	Mar 18
New Zealand	0.912	0.000	-0.018	0.000	106.544	0.000	0.966	52	1567	1507	3.83	1497	4.47	Mar 22
Slovenia	1.095	0.000	-0.016	0.000	44.253	0.000	0.992	68	1502.4	1503	-0.04	1467	2.35	Mar 13

	Guinea-Bissau	0.938	0.000	-0.017	0.000	83.949	0.000	0.992	57	1446.6	1492	-3.14	NA	NA	Apr 29
	Kosovo	1.040	0.000	-0.010	0.000	28.479	0.000	0.975	105	1269.5	1486	-17.05	NA	NA	Mar 31
	Zambia	1.958	0.000	-0.046	0.000	5.351	0.012	0.976	43	1175.5	1412	-20.12	1200	-2.09	Apr 30
	Sierra Leone	1.025	0.000	-0.003	0.353	24.643	0.000	0.992	395	4051.3	1249	69.17	NA	NA	Apr 28
	Latvia	0.763	0.000	-0.007	0.000	65.346	0.000	0.998	116	1141.7	1104	3.3	NA	NA	Mar 20
	Tunisia	0.799	0.000	-0.012	0.000	84.489	0.000	0.994	66	1078.2	1128	-4.62	1068	0.95	Mar 24
	Cyprus	0.956	0.000	-0.014	0.000	45.077	0.000	0.988	66	953.2	985	-3.34	939	1.49	Mar 23
	Niger	0.554	0.000	-0.006	0.000	139.454	0.000	0.985	97	1009.4	1020	-1.05	NA	NA	Apr 3
	Burkina Faso	0.666	0.000	-0.005	0.000	71.089	0.000	0.995	139	979.1	899	8.18	NA	NA	Mar 24
	Andorra	0.653	0.000	-0.010	0.000	97.947	0.000	0.967	68	804.5	854	-6.15	763	5.16	Mar 22
	Chad	1.018	0.000	-0.017	0.000	39.614	0.000	0.992	60	931.6	854	8.33	NA	NA	May 2
	Uruguay	0.547	0.000	-0.002	0.000	87.856	0.000	0.996	325	1200.8	849	29.3	NA	NA	Mar 20
	Georgia	0.880	0.000	-0.007	0.000	31.211	0.000	0.993	129	931.2	888	4.64	NA	NA	Mar 30
	Diamond Princess	0.396	0.000	-0.006	0.000	216.846	0.000	0.799	62	747.3	712	4.72	712	4.72	Feb 10
	San Marino	1.059	0.000	-0.010	0.000	14.835	0.000	0.977	105	713.4	696	2.44	NA	NA	Mar 15
	Malta	0.655	0.000	-0.004	0.000	50.561	0.000	0.978	157	722.2	662	8.34	NA	NA	Mar 23
	Jamaica	0.667	0.000	-0.011	0.000	73.897	0.000	0.982	62	598.3	626	-4.63	NA	NA	Apr 15
	Tanzania	0.636	0.000	-0.017	0.000	98.758	0.000	0.929	37	522.8	509	2.64	509	2.64	Apr 17
	Malawi	0.934	0.000	-0.039	0.004	63.957	0.000	0.976	24	488.1	572	-17.19	NA	NA	May 25
	Mauritius	0.478	0.000	-0.011	0.000	90.930	0.000	0.923	44	345.7	337	2.52	332	3.97	Mar 28
	Vietnam	0.265	0.000	-0.001	0.099	112.014	0.000	0.952	287	384.1	335	12.78	NA	NA	Mar 22
	Montenegro	0.390	0.000	-0.009	0.000	110.891	0.000	0.980	45	331.1	333	-0.57	324	2.16	Mar 31
	Brunei	0.095	0.000	-0.002	0.000	105.432	0.000	0.954	57	141	141	0	141	-0.03	Mar 24
	Cambodia	0.056	0.000	-0.001	0.000	103.154	0.000	0.915	79	124.4	128	-2.89	NA	NA	Mar 29
	Trinidad and Tobago	0.047	0.000	-0.001	0.000	101.467	0.000	0.954	53	116.6	123	-5.49	116	0.55	Apr 4
Mean		1.494	0.000	-0.018	0.000	84.207	0.001	0.981	94.8	25992.1	24515.3	8.6	NA	NA	NA
Standard Errors		0.107	0.000	0.001	0.000	25.523	0.001	0.004	6.4	6403.9	6206.5	1.5	NA	NA	NA

***These models (in shaded cells) were treated as** *failed* fitting cases because the *P*-value for some parameter(s) slightly >0.05, indicating unreliable parameter estimation. The failed cases were excluded from the calculation of the mean and standard error.

Table S10. #The Fisher Information (FI) metric for detecting tipping points: computed for each time windows (based on the same datasets used for PLEC-ITR modeling in Table S7 & S8)*

**City/Province/ China/Worldwide**		**Time Windows**								
		1	***2***	***3***	***4***	***5***	… …	***23***	***24***	***25***
Hubei Province	Wuhan	4	4	4	4	4	… …	4	4	4
	Xiaogan	4	4	4	4	4	… …	4	4	4
	Huanggang	4	4	4	4	4	… …	4	4	4
	Jingzhou	4	4	4	4	4	… …	4	4	4
	Ezhou	4	4	4	4	4	… …	4	4	4
	Suizhou	4	4	4	4	4	… …	4	4	4
	Xiangyang	4	4	4	4	4	… …	4	4	4
	Huangshi	4	4	4	4	4	… …	4	4	4
	Yichang	4	4	4	4	4	… …	4	4	4
	Jingmen	4	4	4	4	4	… …	4	4	4
	Xianning	4	4	4	4	4	… …	4	4	4
	Shiyan	4	4	4	4	4	… …	4	4	4
	Xiantao	4	4	4	4	4	… …	4	4	4
	Tianmen	4	4	4	4	4	… …	4	4	4
	Enshi	4	4	4	4	4	… …	4	4	4
	Qianjiang	4	4	4	4	4	… …	4	4	4
	Shennongjia	4	4	4	4	4	… …	4	4	4
China	Hubei	4	4	4	4	4	… …	4	4	4
	Guangdong	4	4	4	4	4	… …	4	4	4
	Henan	4	4	4	4	4	… …	4	4	4
	Zhejiang	4	4	4	4	4	… …	4	4	4
	Hunan	4	4	4	4	4	… …	4	4	4
	Anhui	4	4	4	4	4	… …	4	4	4
	Jiangxi	4	4	4	4	4	… …	4	4	4
	Shandong	4	4	4	4	4	… …	4	4	4
	Jiangsu	4	4	4	4	4	… …	4	4	4
	Chongqing	4	4	4	4	4	… …	4	4	4
	Sichuan	4	4	4	4	4	… …	4	4	4
	Heilongjiang	4	4	4	4	4	… …	4	4	4
	Beijing	4	4	4	4	4	… …	4	4	4
	Shanghai	4	4	4	4	4	… …	4	4	4
	Hebei	4	4	4	4	4	… …	4	4	4
	Fujian	4	4	4	4	4	… …	4	4	4
	Guangxi	4	4	4	4	4	… …	4	4	4
	Shaanxi	4	4	4	4	4	… …	4	4	4
	Yunnan	4	4	4	4	4	… …	4	4	4
	Hainan	4	4	4	4	4	… …	4	4	4
	Guizhou	4	4	4	4	4	… …	4	4	4
	Tianjin	4	4	4	4	4	… …	4	4	4
	Shanxi	4	4	4	4	4	… …	4	4	4
	Liaoning	4	4	4	4	4	… …	4	4	4
	HongKong	4	4	4	4	4	… …	4	4	4
	Jilin	4	4	4	4	4	… …	4	4	4

	Gansu	4	4	4	4	4	... ...	4	4	4
	Xinjiang	4	4	4	4	4	... ...	4	4	4
	Neimenggu	4	4	4	4	4	... ...	4	4	4
	Ningxia	4	4	4	4	4	... ...	4	4	4
	Taiwan	4	4	4	4	4	... ...	4	4	4
	Qinghai	4	4	4	4	4	... ...	4	4	4
	Macao	4	4	4	4	4	... ...	4	4	4
	Xizang	NA	NA	NA	NA	NA	NA	NA	NA	NA
China		4	4	4	4	4	... ...	4	4	4
World		4	4	4	4	4	... ...	4	4	4

All the FI values=4 (maximum), suggested the failure for detection of any tipping points.

#Prediction of tipping points with Fisher Information (FI)

Section 4.5 in the main text presented a simple introduction on the application of Fisher information for detecting the tipping points. Table S10 above exhibited the computational results from applying (Sundstrom *et al*. 2017) framework. As shown in Table S7, virtually all Fisher Information (FI) values at every time window of the COVID-19 time series data (we tried) equal *4*, which indicates no existence of tipping points. This additional exploratory analysis not only revealed the difficulty of the problem for detecting the inflection points, but also suggested the value of PLEC-ITR model.

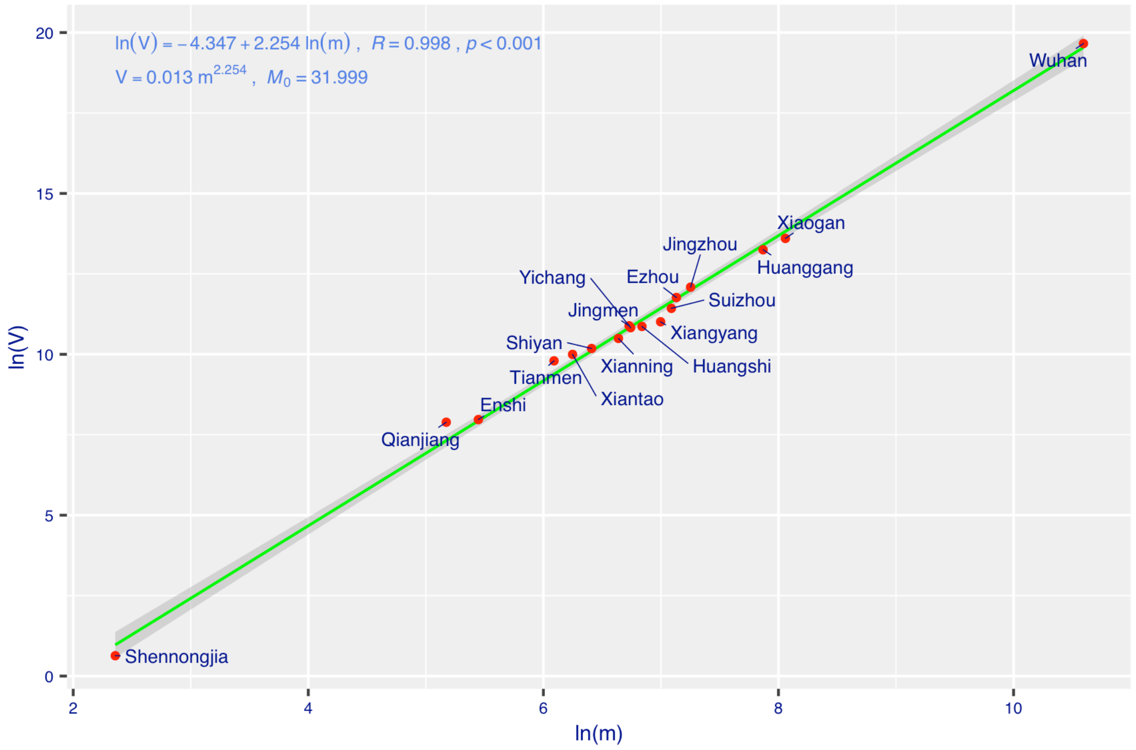

Fig S1A. TPL (Taylor's power law) model fitted to the cumulative infections of COVID-19 in the Hubei province of China (The data collection ending date=June 12th)

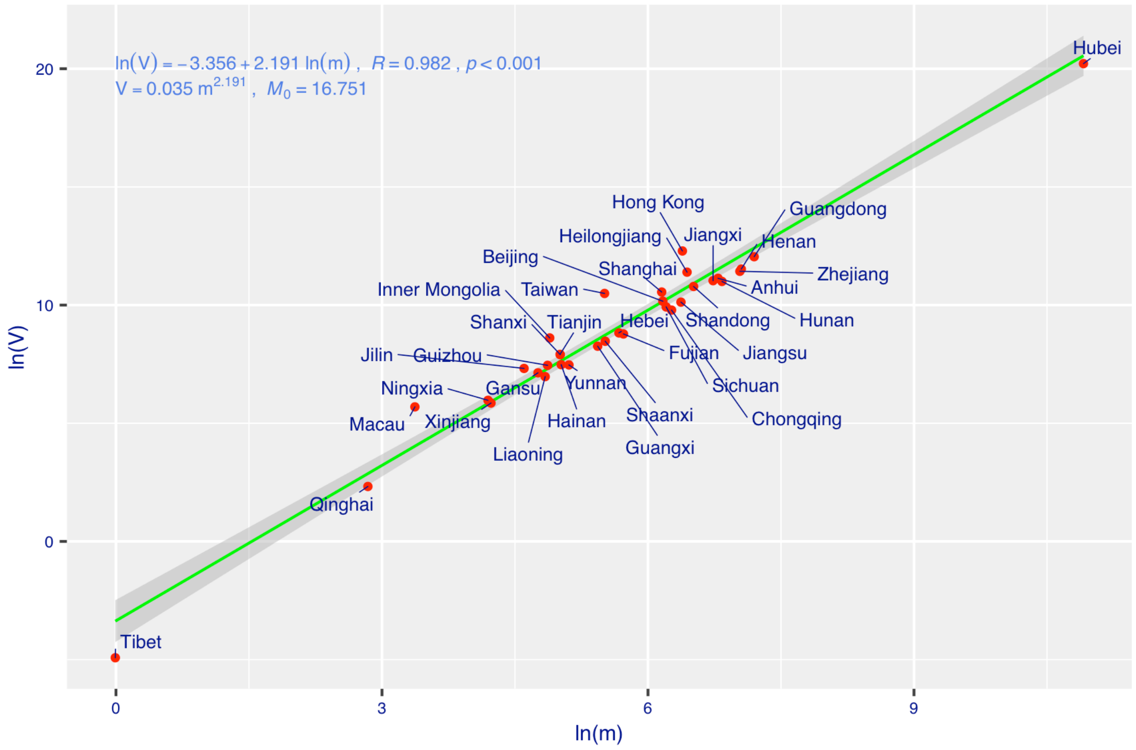

Fig S1B. TPL (Taylor's power law) model fitted to the cumulative infections of COVID-19 in China (The data collection ending date=June 12th)

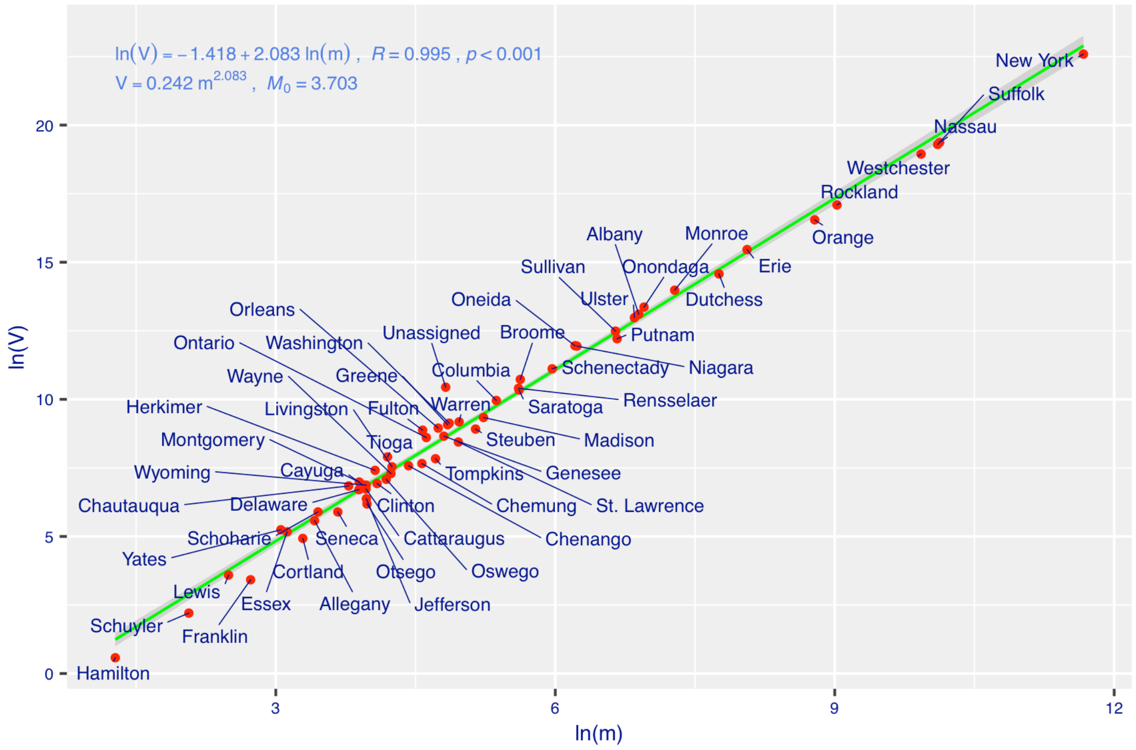

Fig S1C. TPL (Taylor's power law) model fitted to the cumulative infections of COVID-19 in New York State of the USA (The data collection ending date=June 12th)

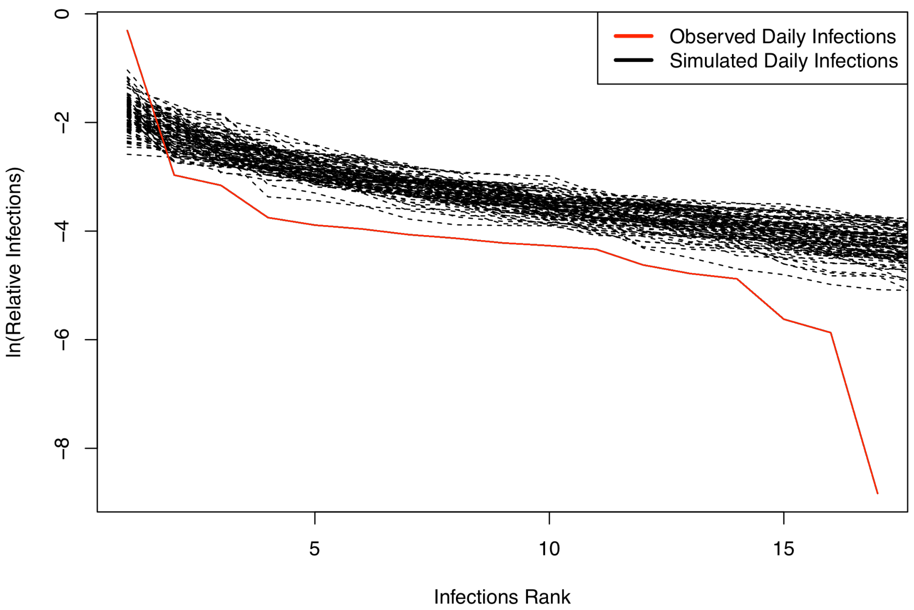

Fig S2A. Approximating the metapopulation of daily incremental COVID-19 infections in Hubei province of China with Hubbell's (2001) UNTB, implemented with Harris *et al*. (2017) HDP-MSN model. (The data collection ending date=June 12th)

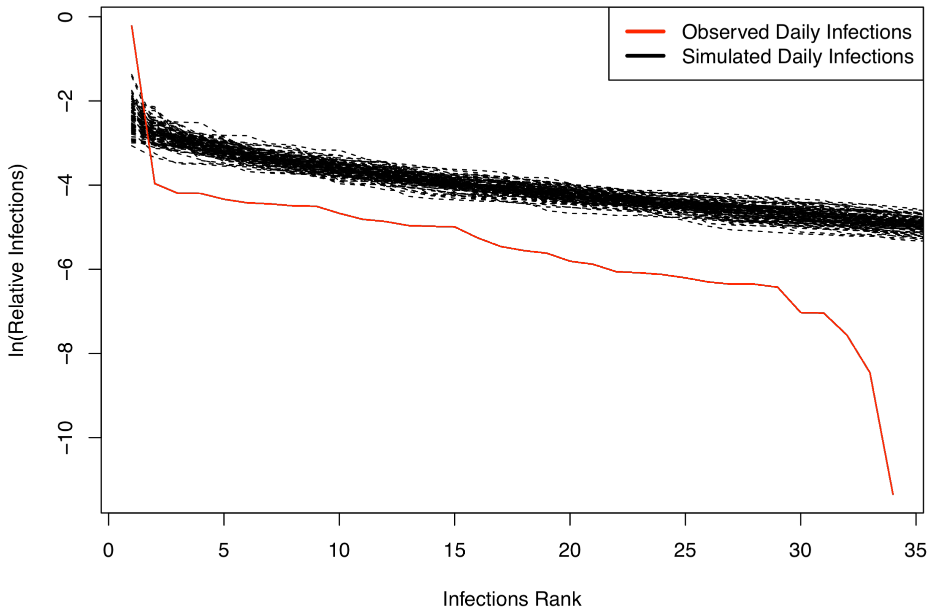

Fig S2B. Approximating the metapopulation of daily incremental COVID-19 infections in China with Hubbell's (2001) UNTB, implemented with Harris *et al*. (2017) HDP-MSN model. (The data collection ending date=June 12th)

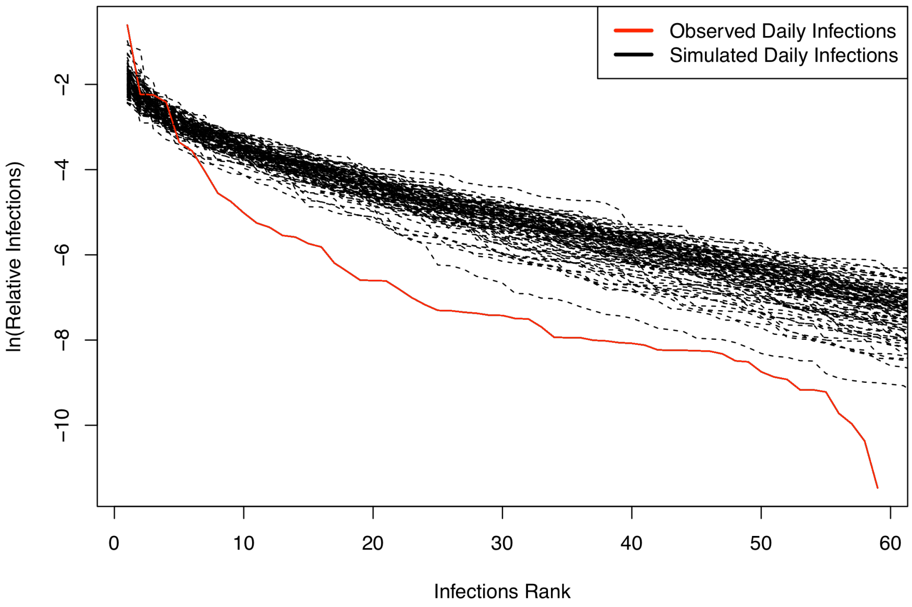

Fig S2C. Approximating the metapopulation of daily incremental COVID-19 infections in New York State of the USA with Hubbell's (2001) UNTB, implemented with Harris *et al*. (2017) HDP-MSN model. (The data collection ending date=June12th)

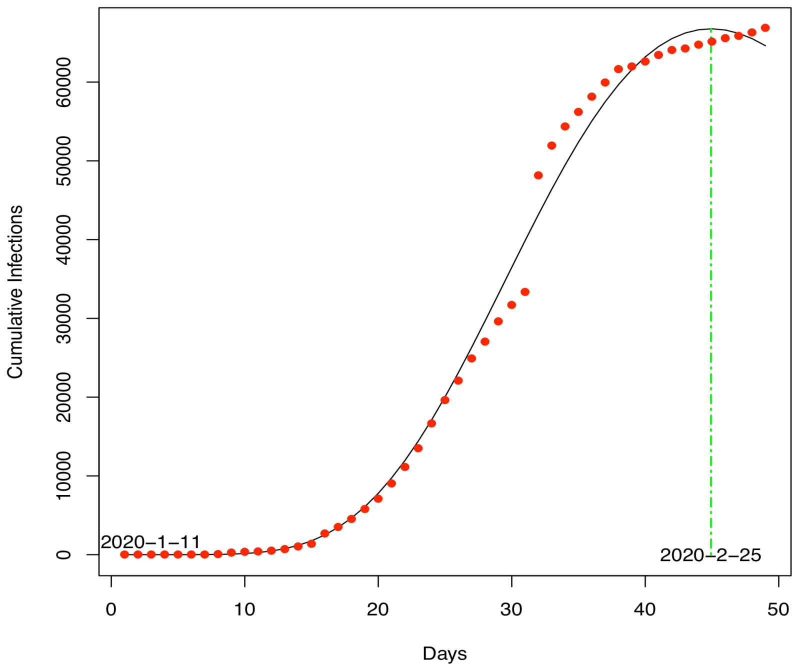

Fig S3A. The PLEC-ITR model fitted to the COVID-19 infections in Hubei Province of China (X-axis: Day 1=January 11th 2020): the dots represent for observed infection numbers and the curve for the PLEC model. The datasets used are the same as in Tables S7 & S8.

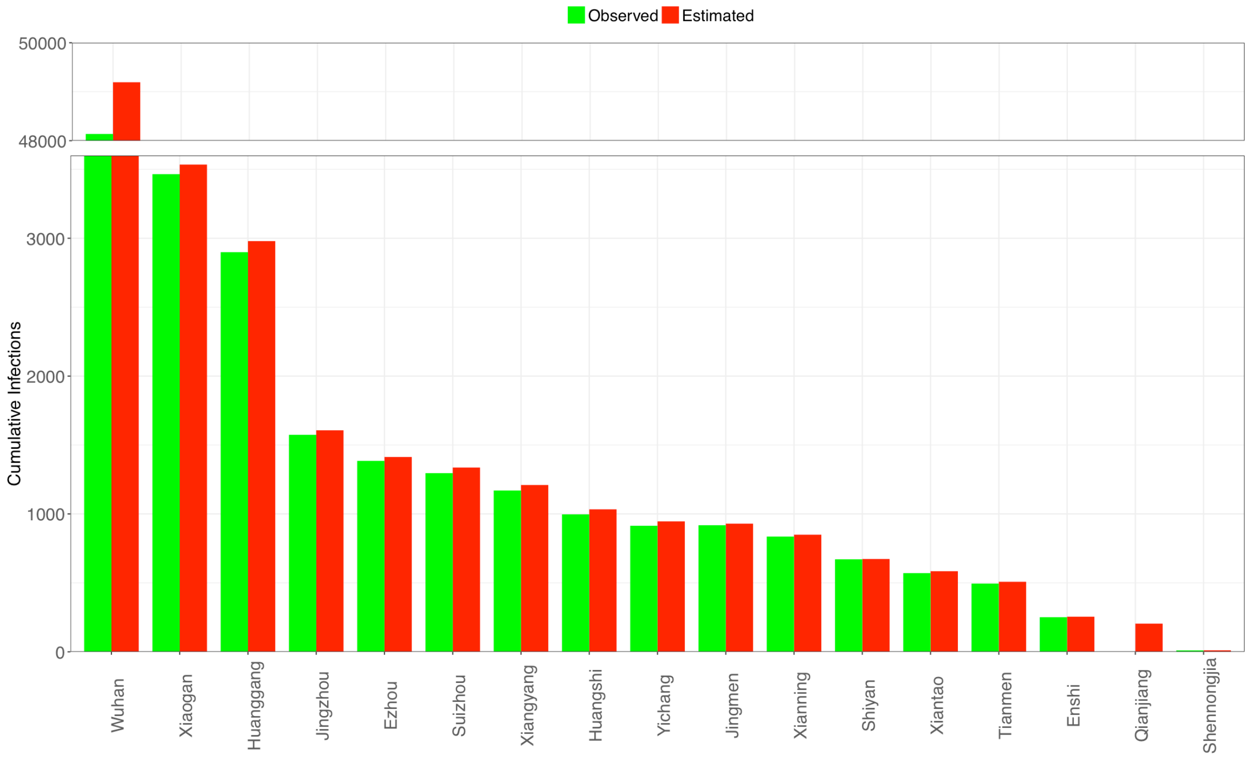

Fig S3B. Comparisons of the observed and estimated maximal infection numbers (I_{max}) for the 17 cities of Hubei Province, China (The datasets used are the same as in Tables S7 & S8)

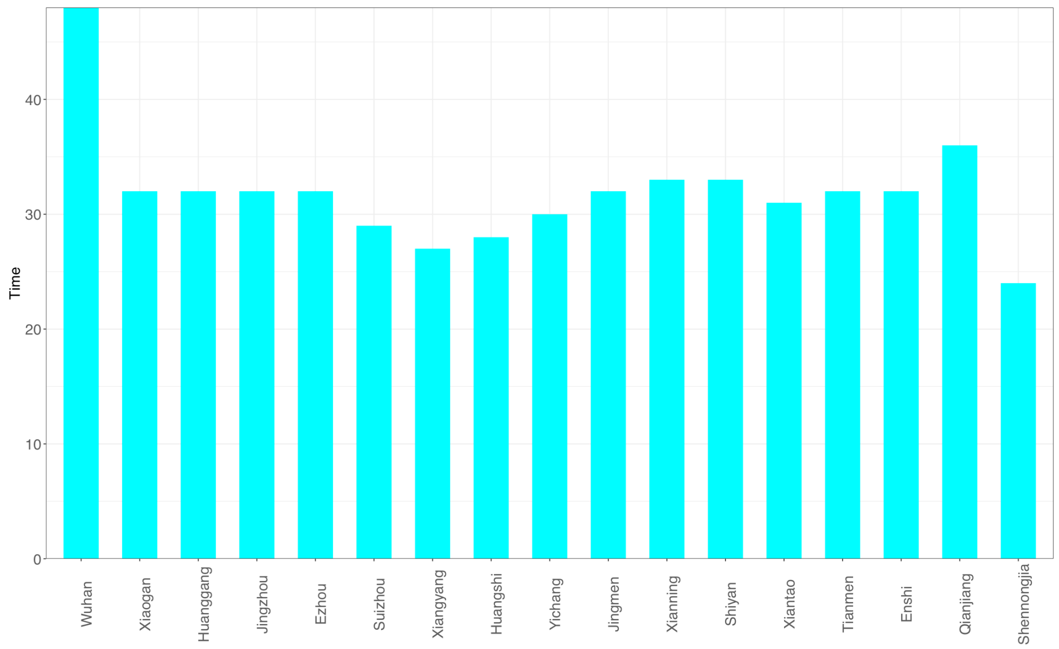

Fig S3C. Estimated inflection time point (T_{max}) of COVID-19 infections for the 17 cities of Hubei Province, China (The datasets used are the same as in Tables S7 & S8.)
